# Supplementary material for: GenomeScope 2.0 and Smudgeplot for reference-free profiling of polyploid genomes
Source: Nat Commun. 2020 Mar 18;11:1432. doi: 10.1038/s41467-020-14998-3 (PMC7080791; doi:10.1038/s41467-020-14998-3)
Supplement: Supplementary file 1 — Supplementary Information [file 41467_2020_14998_MOESM1_ESM.pdf]

GenomeScope 2.0 and Smudgeplot for reference-free profiling of polyploid genomes

Supplementary Information

Ranallo-Benavidez et al.

Supplementary Figures

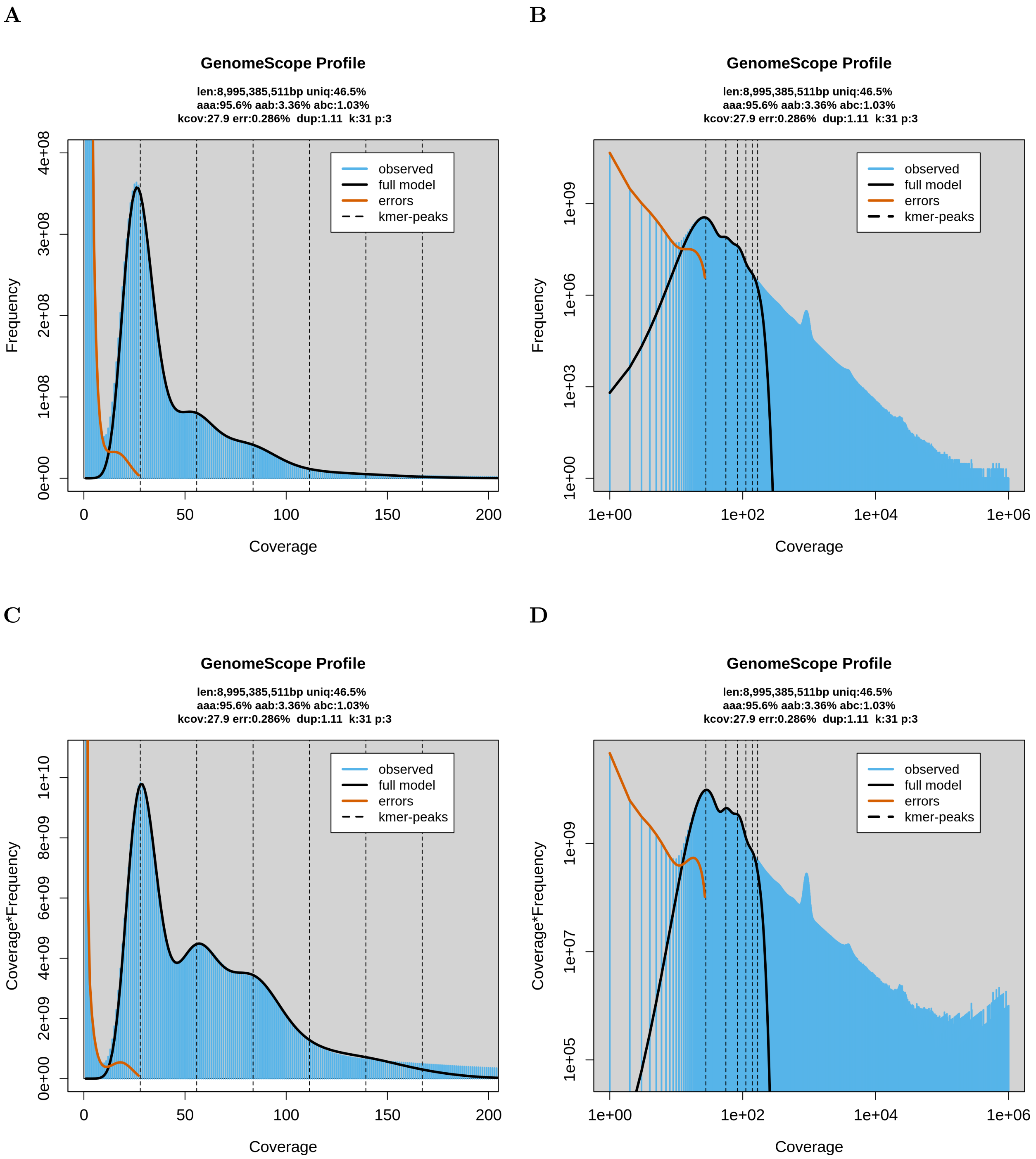

Supplementary Figure 1: GenomeScope coastal redwood results (*Sequoia sempervirens*). Plots of the best fit model overlaying the k-mer spectrum for (A) untransformed linear, (B) untransformed log, (C) transformed linear, and (D) transformed log. While the coastal redwood is hexaploid, these data are triploid since they come from the megagametophyte extracted from a seed.

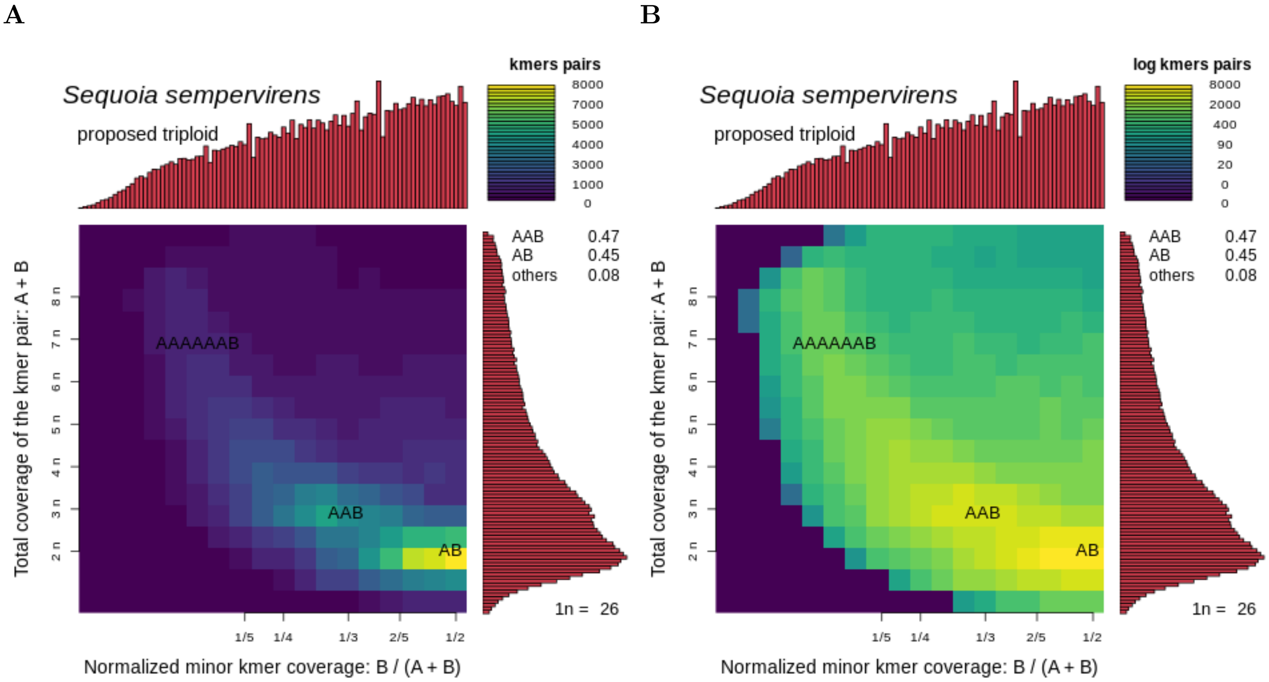

Supplementary Figure 2: Smudgeplot coastal redwood results (*Sequoia sempervirens*). Smudgeplots are shown using either (A) a linear scale or (B) a log scale. The coloration indicates the approximate number of k-mer pairs per bin.

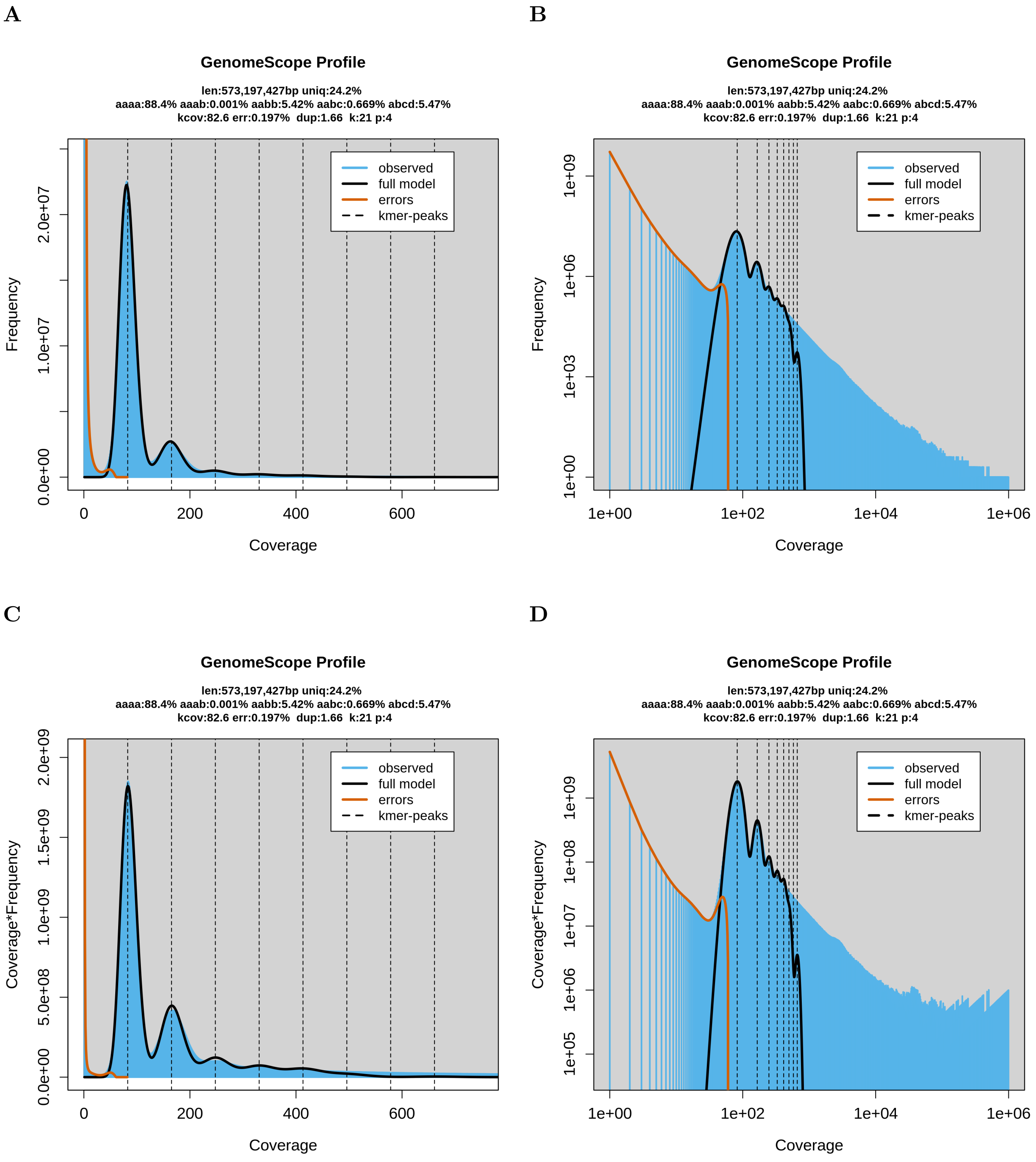

Supplementary Figure 3: GenomeScope cotton results (*Gossypium barbadense*). Plots of the best fit model overlaying the k-mer spectrum for (A) untransformed linear, (B) untransformed log, (C) transformed linear, and (D) transformed log.

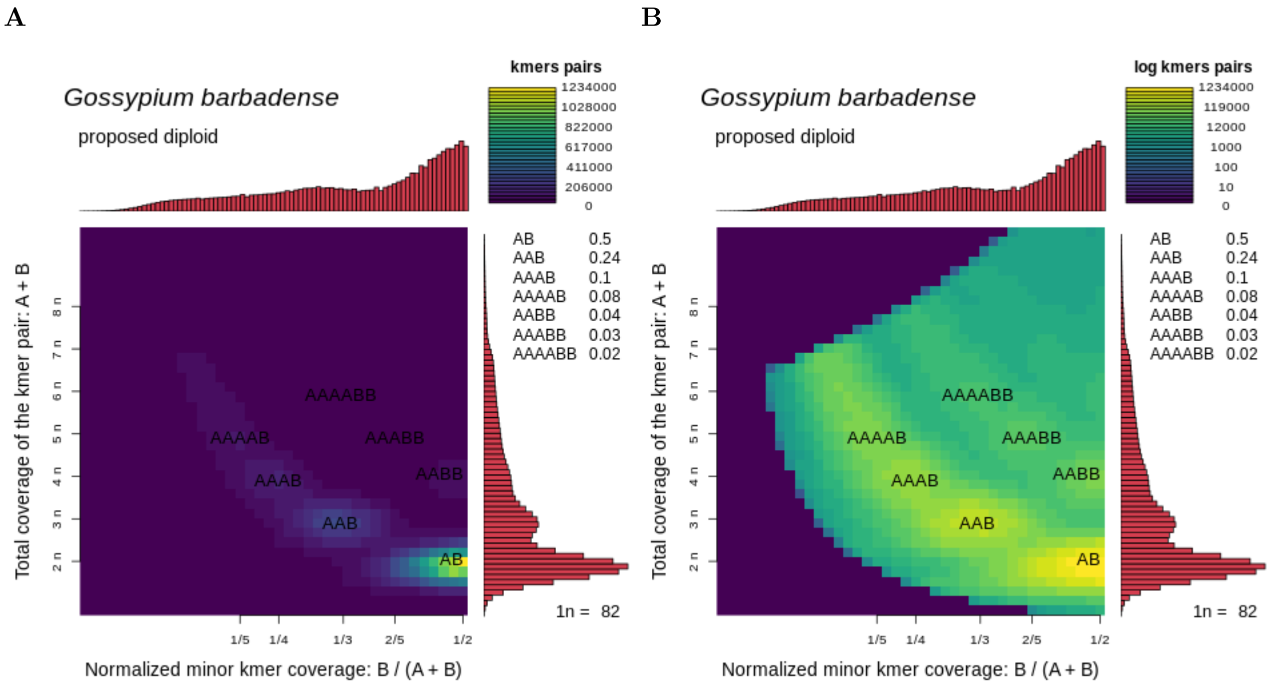

Supplementary Figure 4: Smudgeplot cotton results (*Gossypium barbadense*). Smudgeplots are shown using either (A) a linear scale or (B) a log scale. The coloration indicates the approximate number of k-mer pairs per bin.

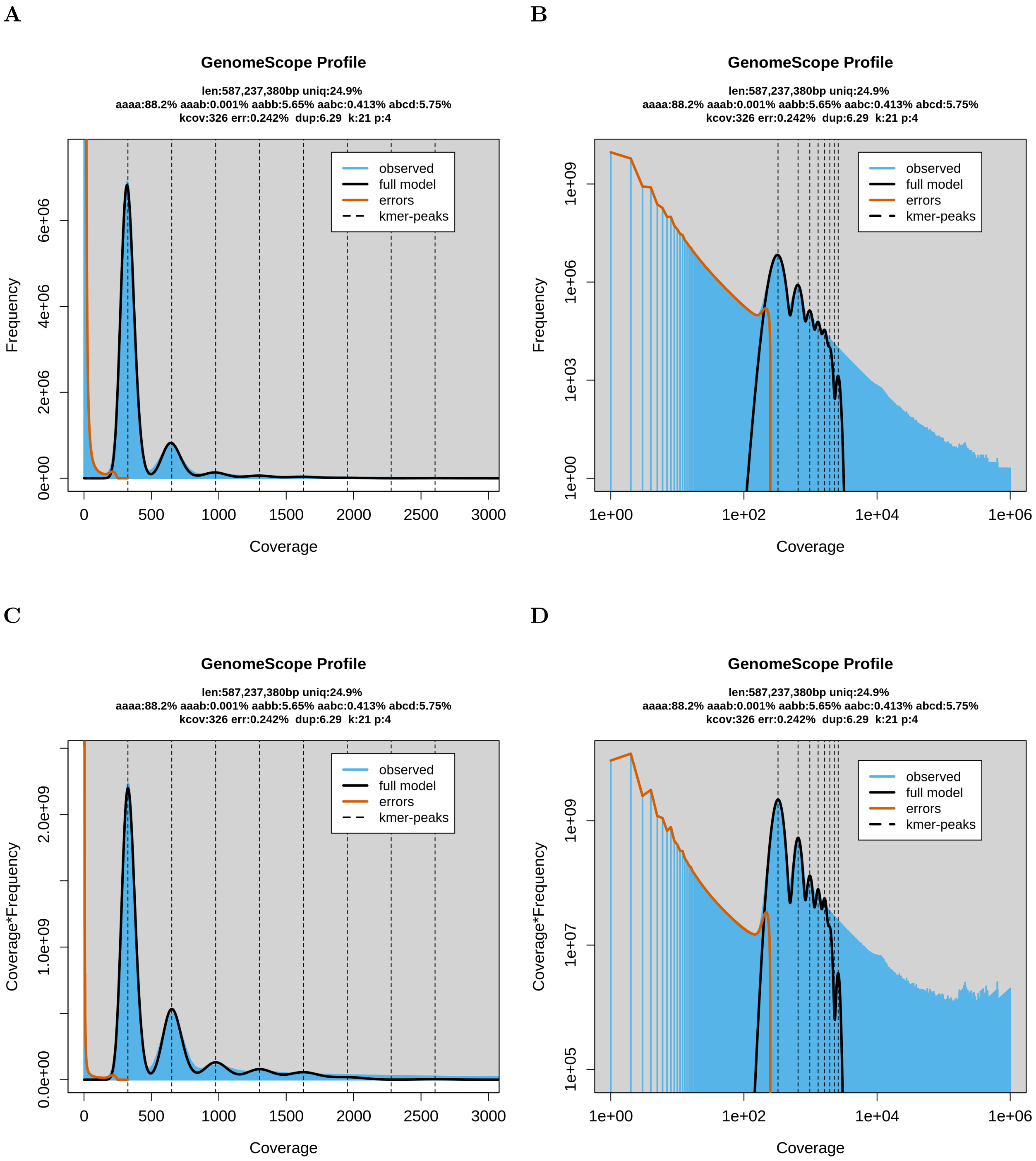

Supplementary Figure 5: GenomeScope cotton results (*Gossypium hirsutum*). Plots of the best fit model overlaying the k-mer spectrum for (A) untransformed linear, (B) untransformed log, (C) transformed linear, and (D) transformed log.

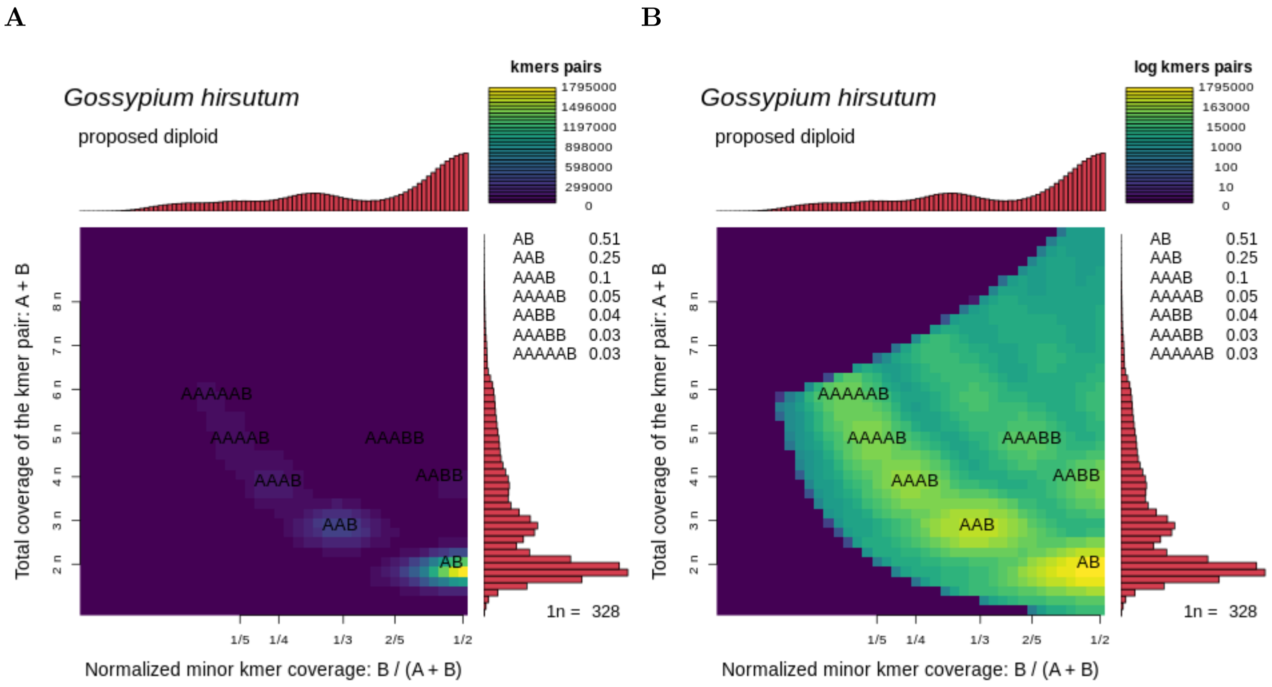

Supplementary Figure 6: Smudgeplot cotton results (*Gossypium hirsutum*). Smudgeplots are shown using either (A) a linear scale or (B) a log scale. The coloration indicates the approximate number of k-mer pairs per bin.

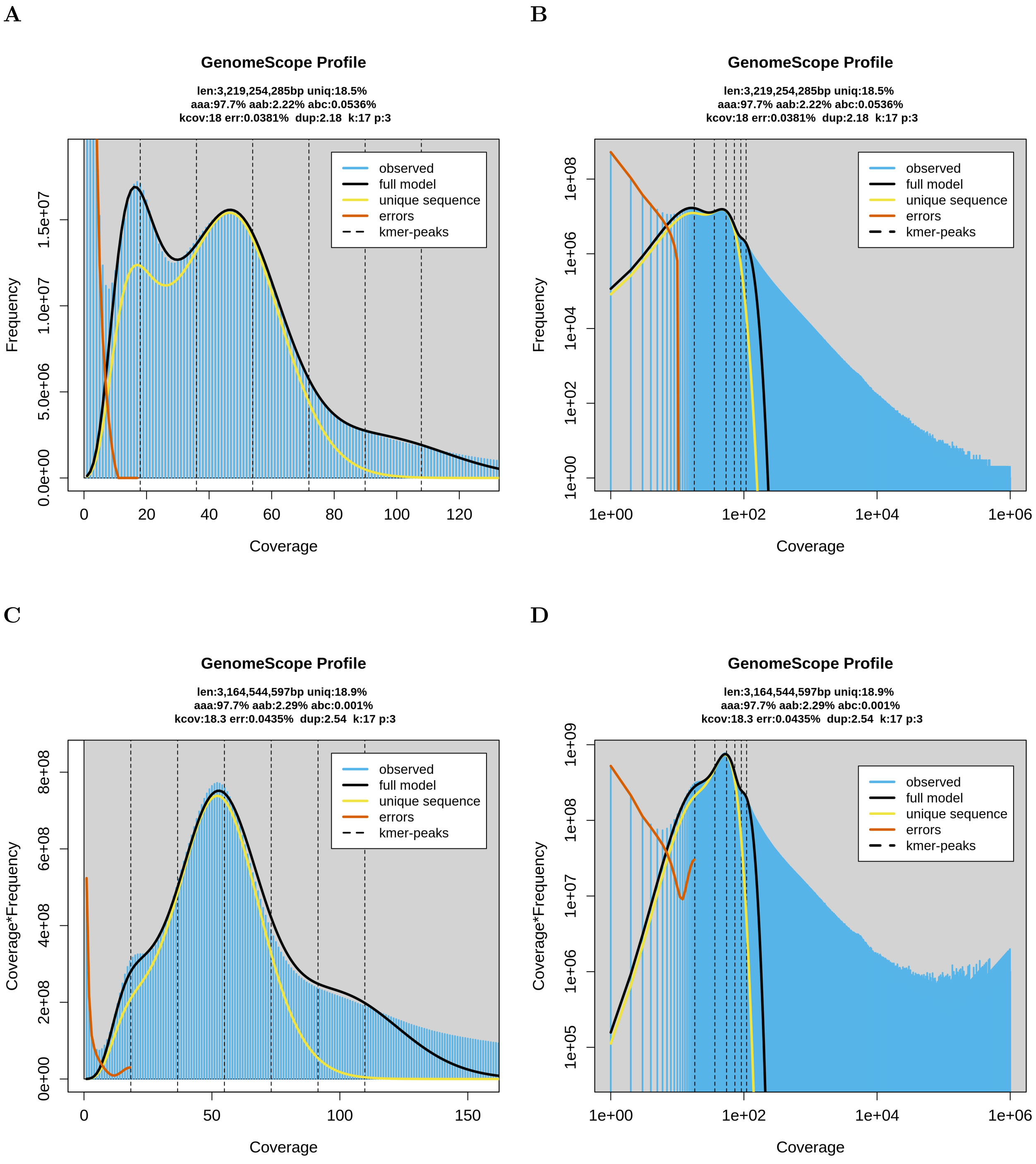

Supplementary Figure 7: GenomeScope marbled crayfish results (*Procambarus virginalis*). Plots of the best fit model overlaying the k-mer spectrum for (A) untransformed linear, (B) untransformed log, (C) transformed linear, and (D) transformed log.

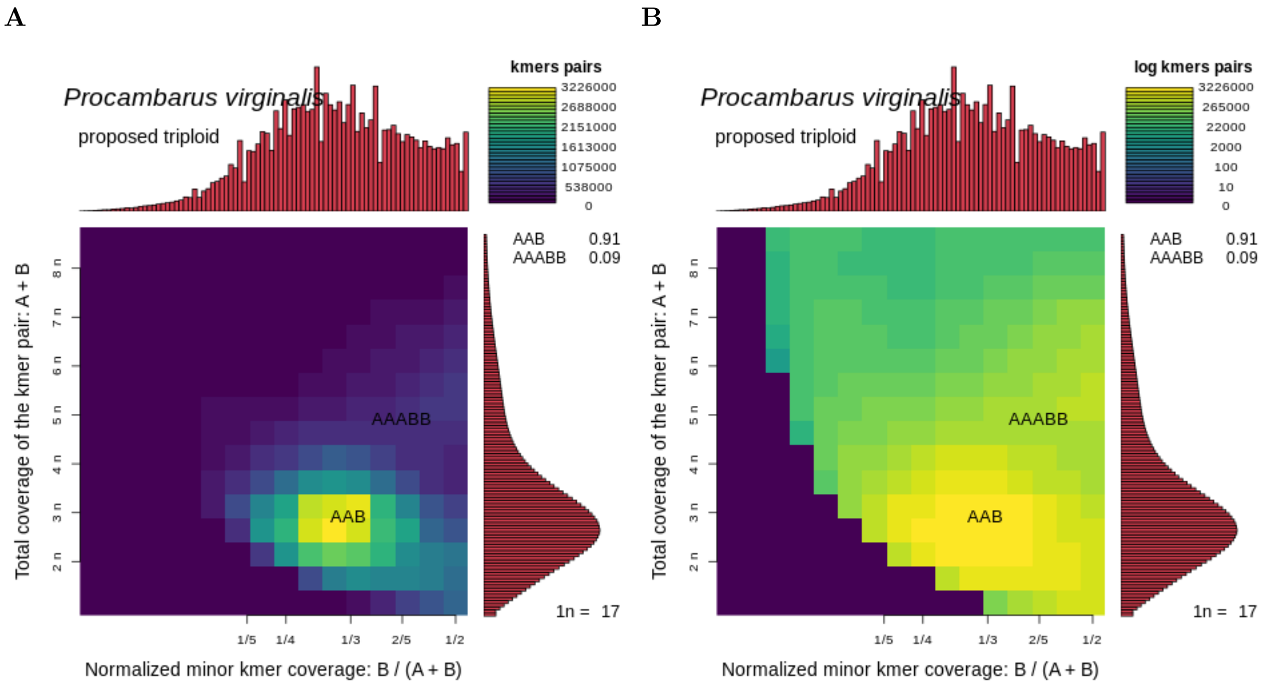

Supplementary Figure 8: Smudgeplot marbled crayfish results (*Procambarus virginalis*). Smudgeplots are shown using either (A) a linear scale or (B) a log scale. The coloration indicates the approximate number of k-mer pairs per bin.

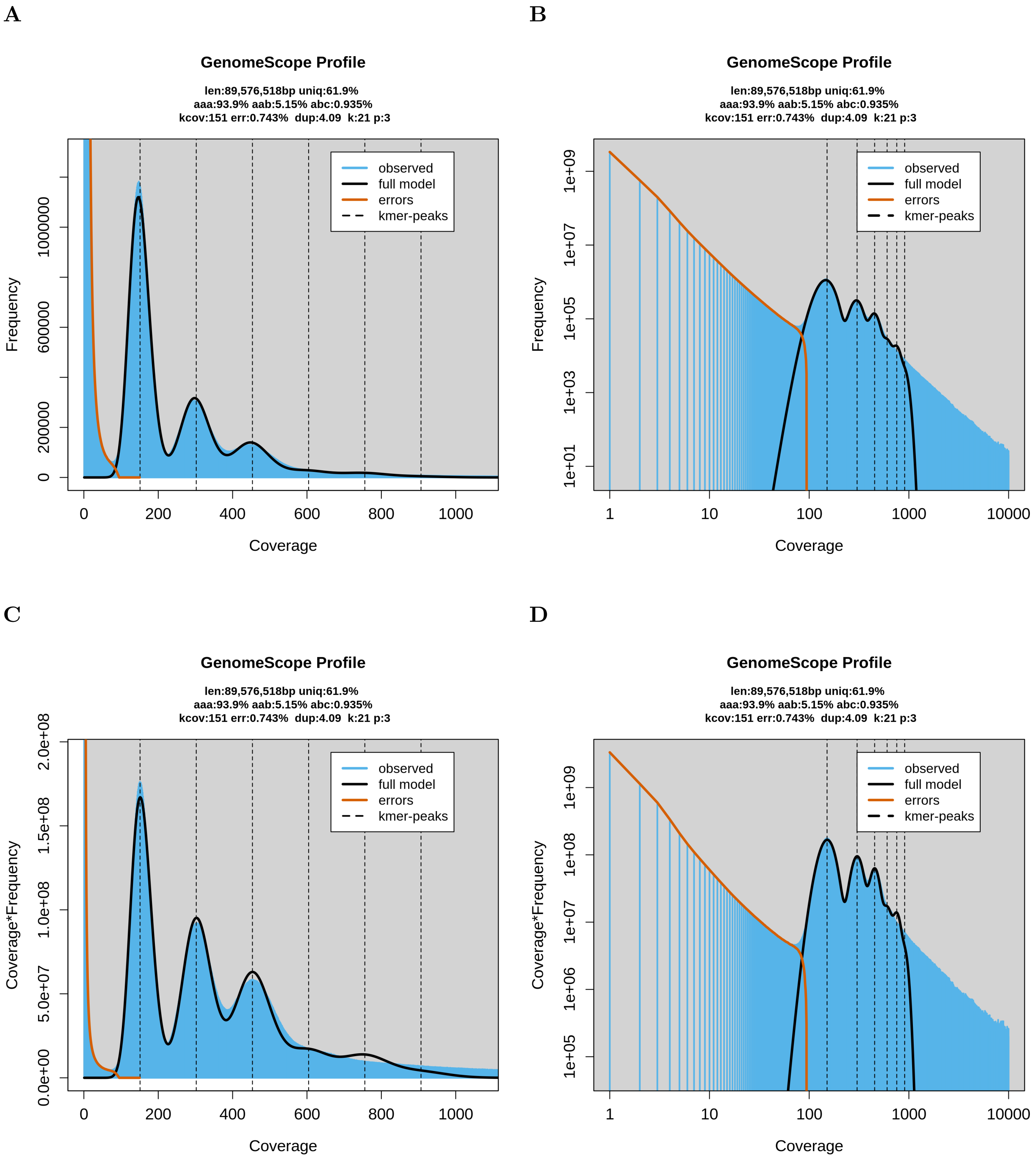

Supplementary Figure 9: GenomeScope root-knot nematode results (*Meloidogyne enterolobii*). Plots of the best fit model overlaying the k-mer spectrum for (A) untransformed linear, (B) untransformed log, (C) transformed linear, and (D) transformed log.

A B

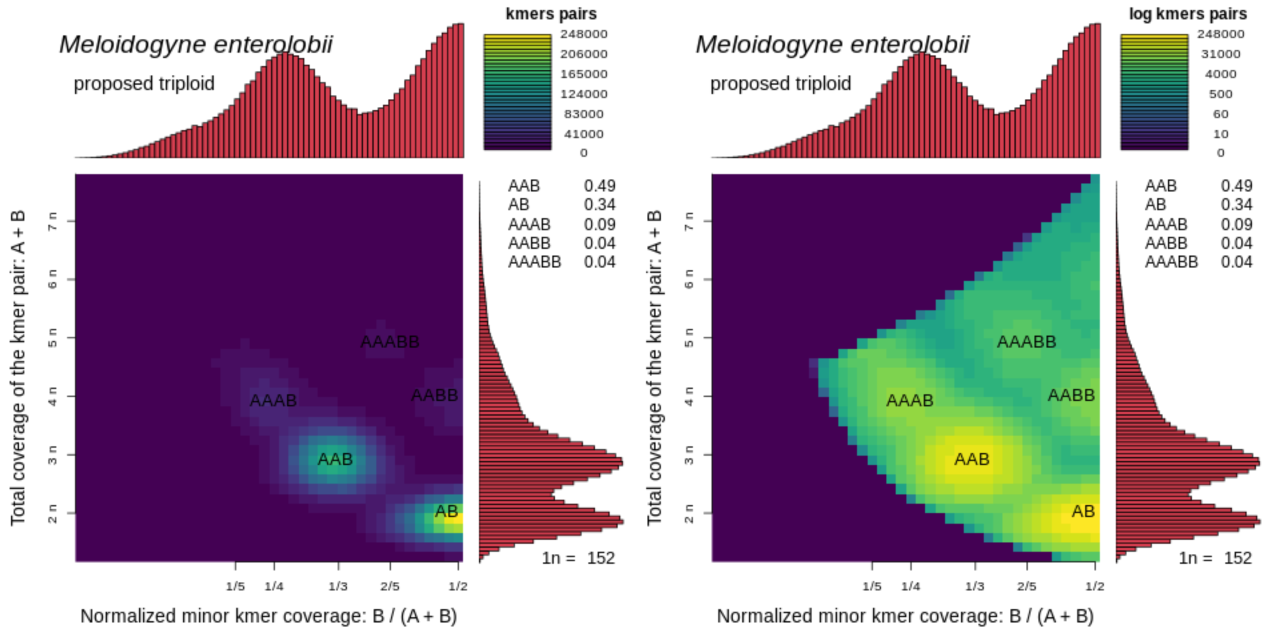

Supplementary Figure 10: Smudgeplot root-knot nematode results (*Meloidogyne enterolobii*). Smudgeplots are shown using either (A) a linear scale or (B) a log scale. The coloration indicates the approximate number of k-mer pairs per bin.

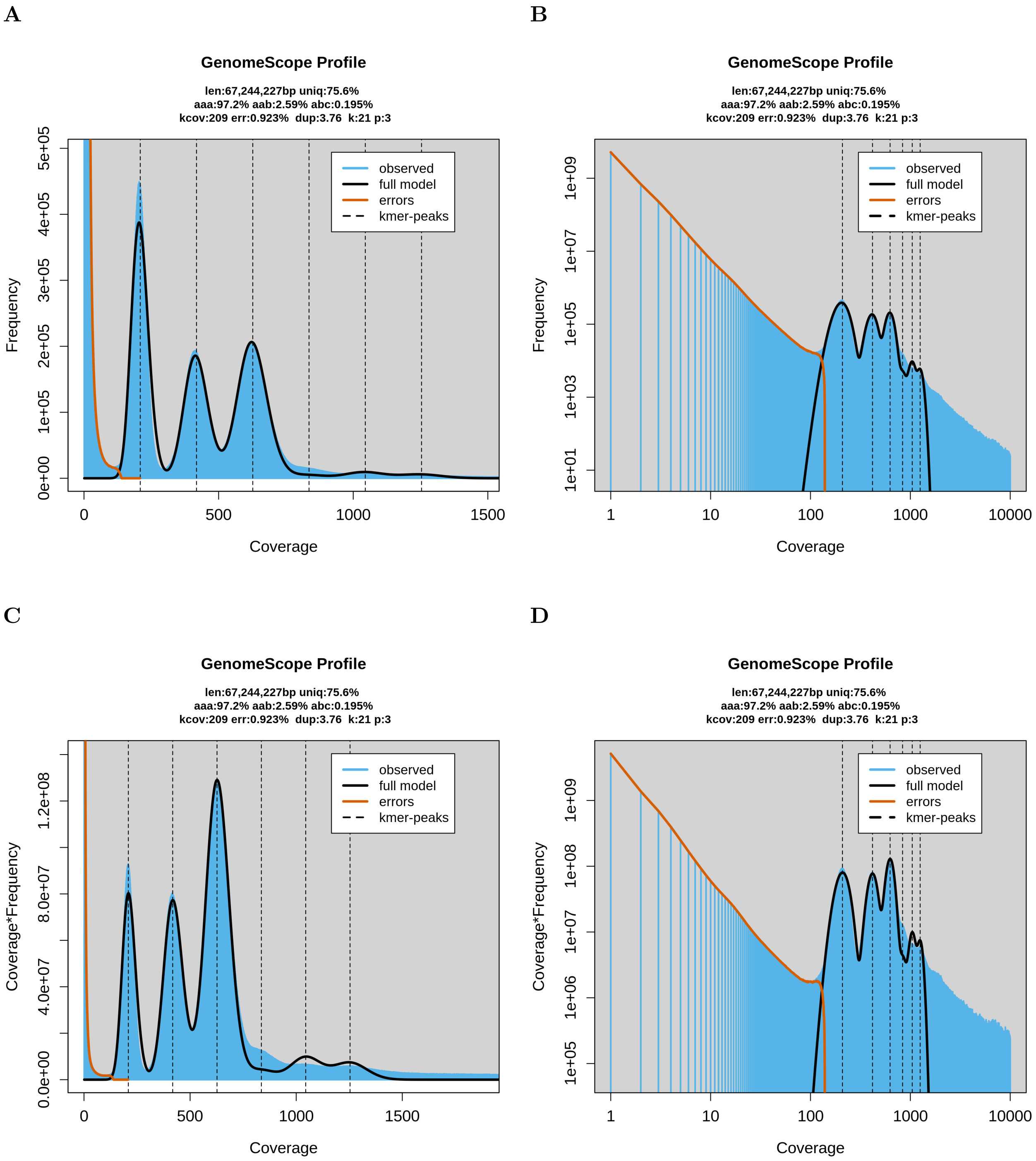

Supplementary Figure 11: GenomeScope root-knot nematode results (*Meloidogyne floridensis*). Plots of the best fit model overlaying the k-mer spectrum for (A) untransformed linear, (B) untransformed log, (C) transformed linear, and (D) transformed log.

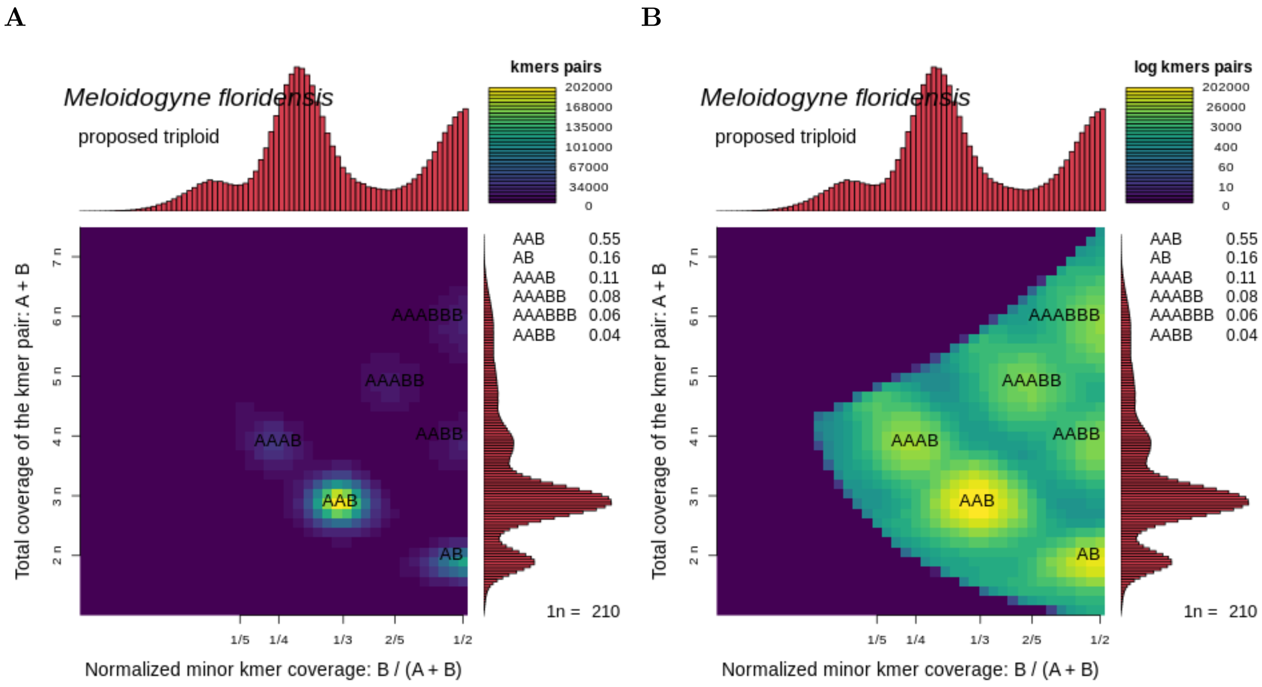

Supplementary Figure 12: Smudgeplot root-knot nematode results (*Meloidogyne floridensis*). Smudgeplots are shown using either (A) a linear scale or (B) a log scale. The coloration indicates the approximate number of k-mer pairs per bin.

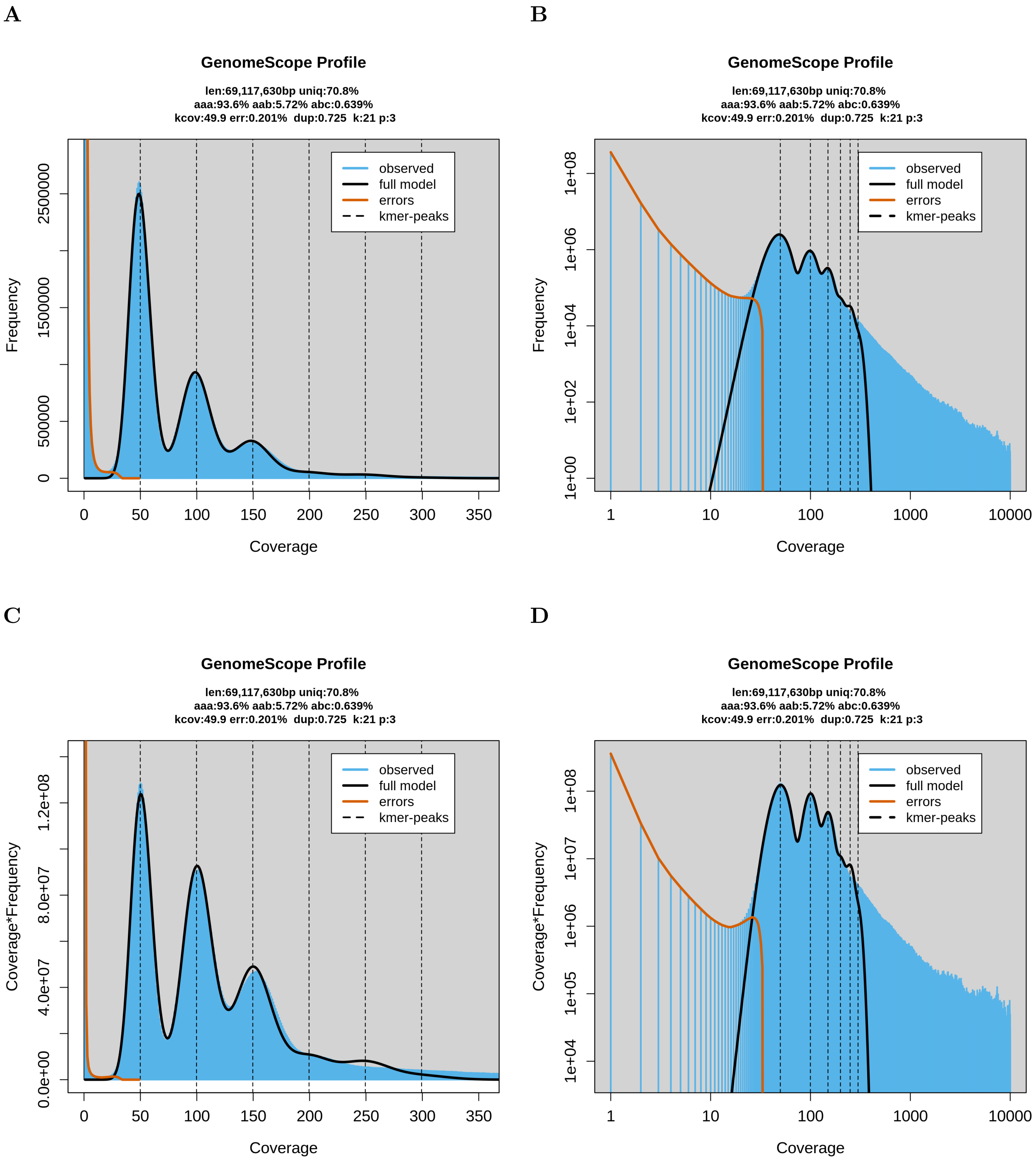

Supplementary Figure 13: GenomeScope root-knot nematode results (*Meloidogyne incognita*). Plots of the best fit model overlaying the k-mer spectrum for (A) untransformed linear, (B) untransformed log, (C) transformed linear, and (D) transformed log.

A B

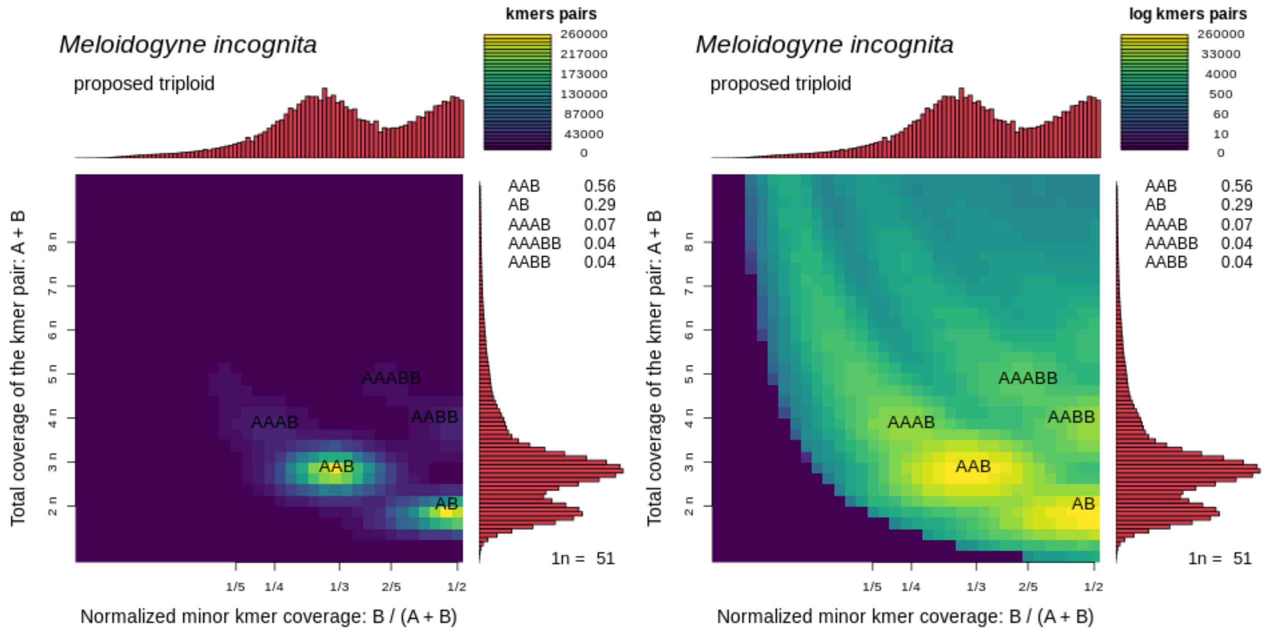

Supplementary Figure 14: Smudgeplot root-knot nematode results (*Meloidogyne incognita*). Smudgeplots are shown using either (A) a linear scale or (B) a log scale. The coloration indicates the approximate number of k-mer pairs per bin.

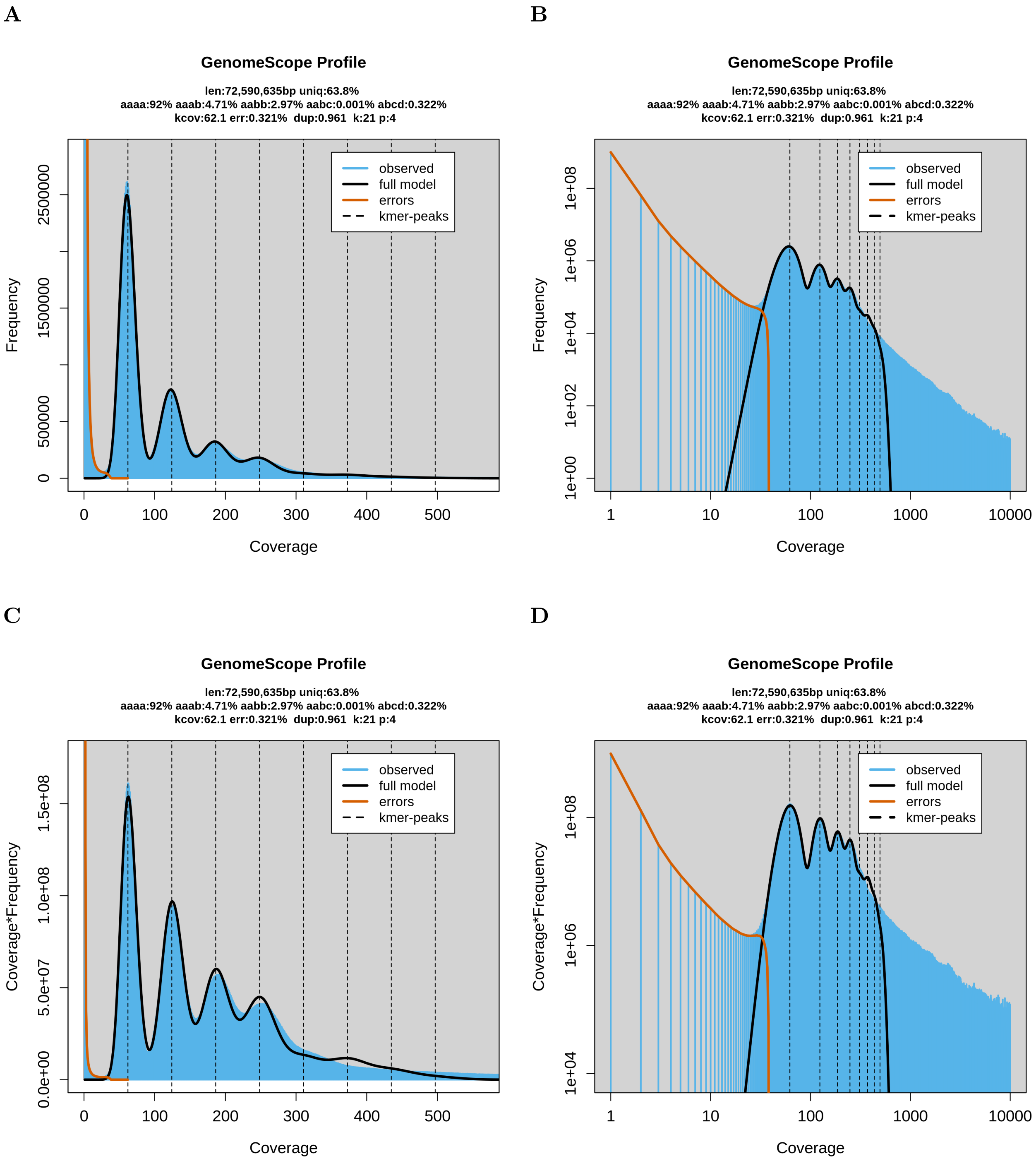

Supplementary Figure 15: GenomeScope root-knot nematode results (*Meloidogyne arenaria*). Plots of the best fit model overlaying the k-mer spectrum for (A) untransformed linear, (B) untransformed log, (C) transformed linear, and (D) transformed log.

A B

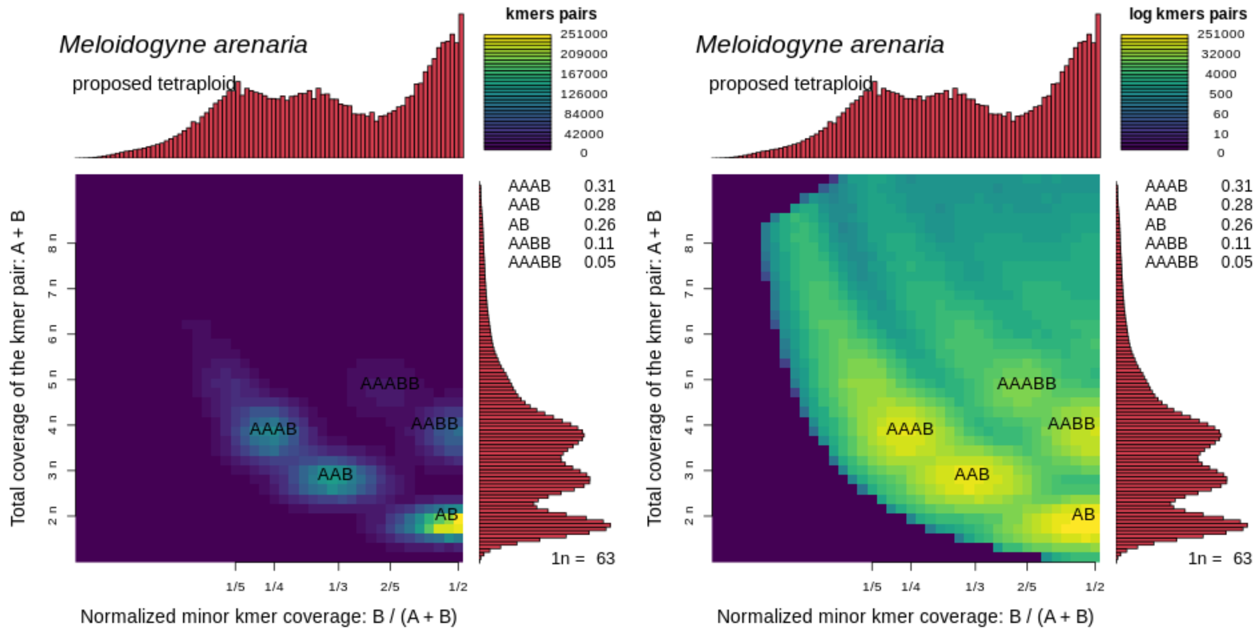

Supplementary Figure 16: Smudgeplot root-knot nematode results (*Meloidogyne arenaria*). Smudgeplots are shown using either (A) a linear scale or (B) a log scale. The coloration indicates the approximate number of k-mer pairs per bin.

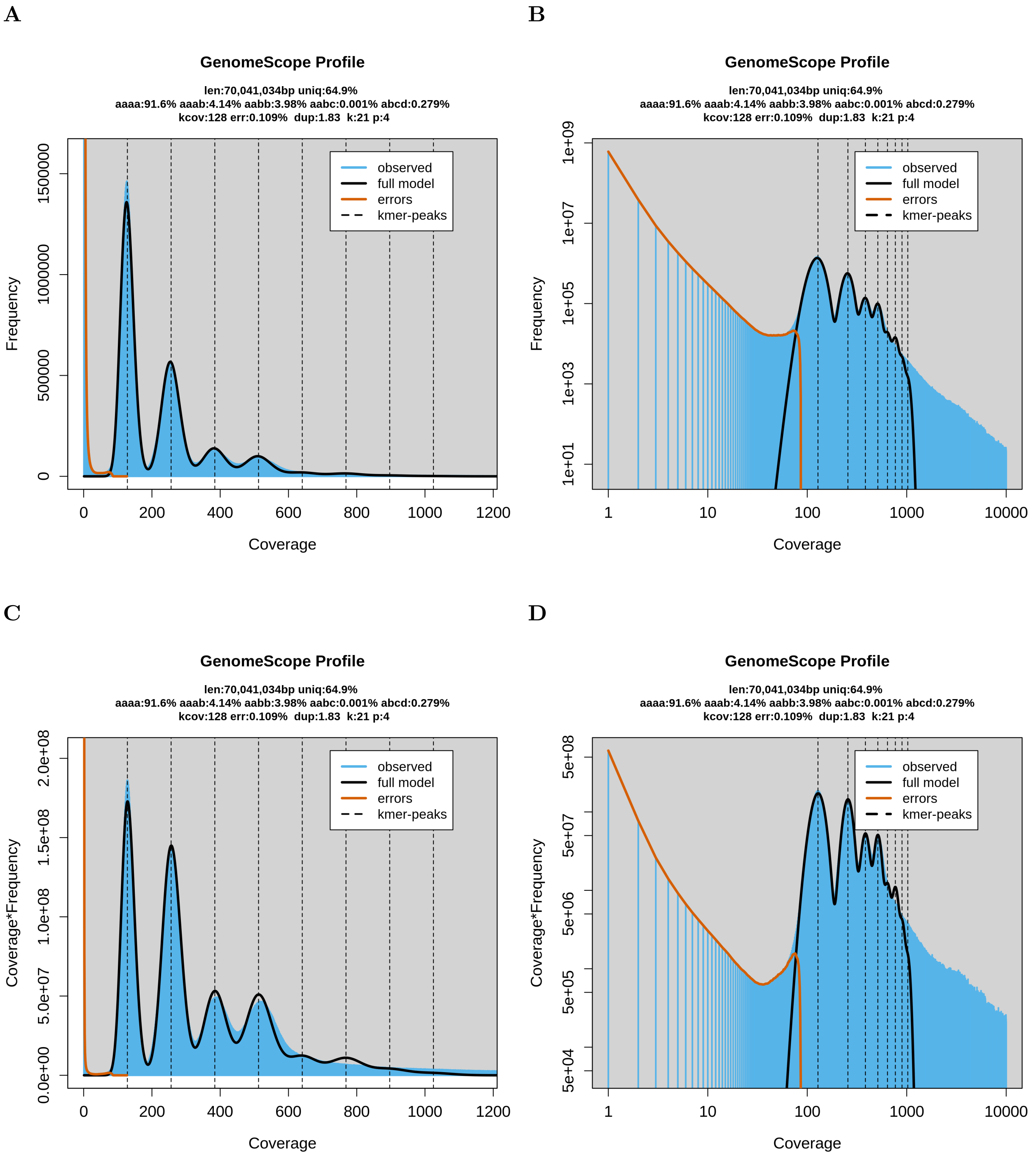

Supplementary Figure 17: GenomeScope root-knot nematode results (*Meloidogyne javanica*). Plots of the best fit model overlaying the k-mer spectrum for (A) untransformed linear, (B) untransformed log, (C) transformed linear, and (D) transformed log.

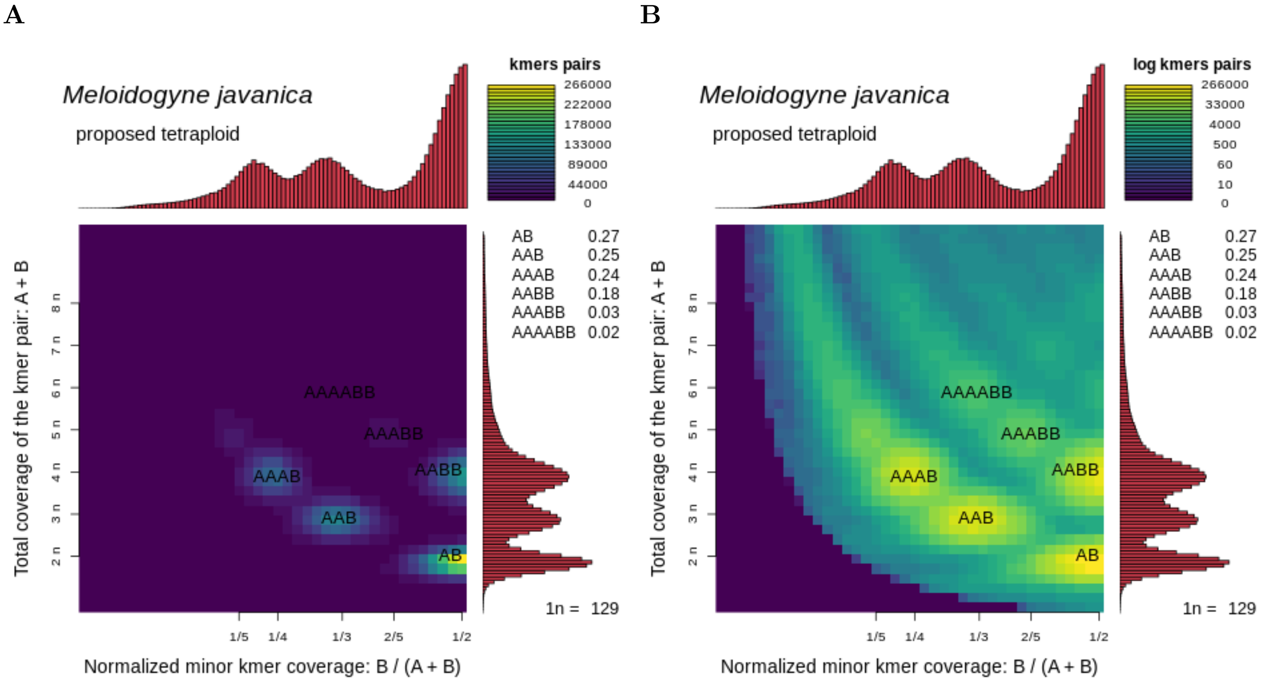

Supplementary Figure 18: Smudgeplot root-knot nematode results (*Meloidogyne javanica*). Smudgeplots are shown using either (A) a linear scale or (B) a log scale. The coloration indicates the approximate number of k-mer pairs per bin.

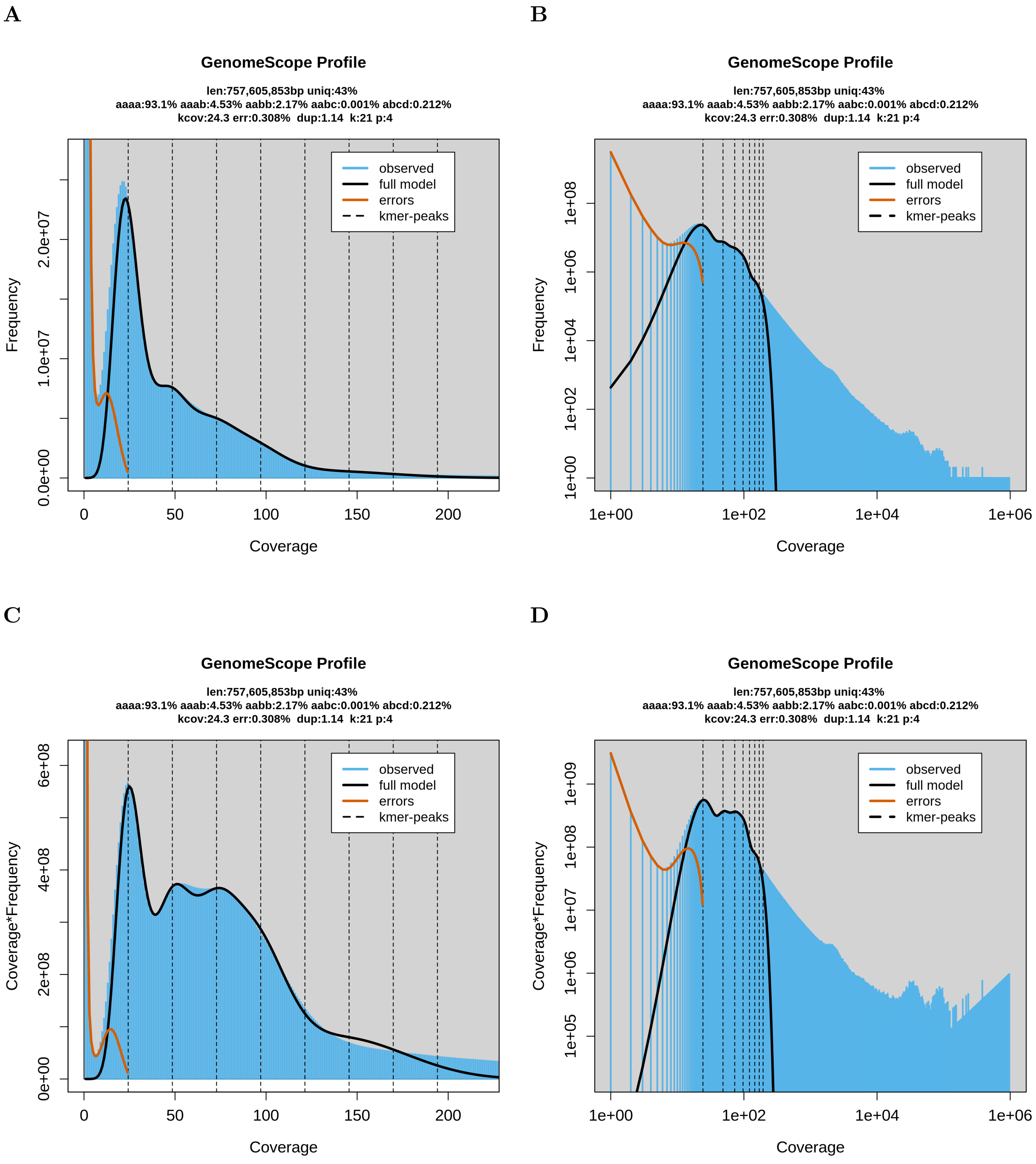

Supplementary Figure 19: GenomeScope potato results (*Solanum tuberosum*). Plots of the best fit model overlaying the k-mer spectrum for (A) untransformed linear, (B) untransformed log, (C) transformed linear, and (D) transformed log.

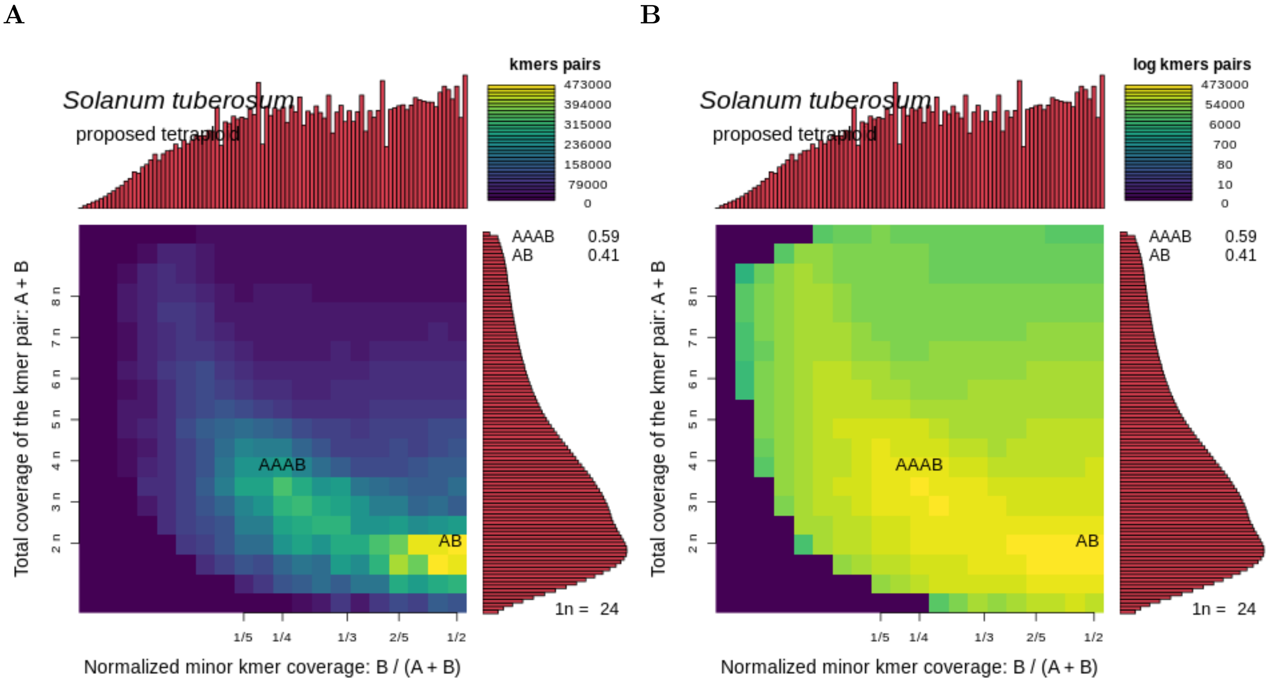

Supplementary Figure 20: Smudgeplot potato results (*Solanum tuberosum*). Smudgeplots are shown using either (A) a linear scale or (B) a log scale. The coloration indicates the approximate number of k-mer pairs per bin.

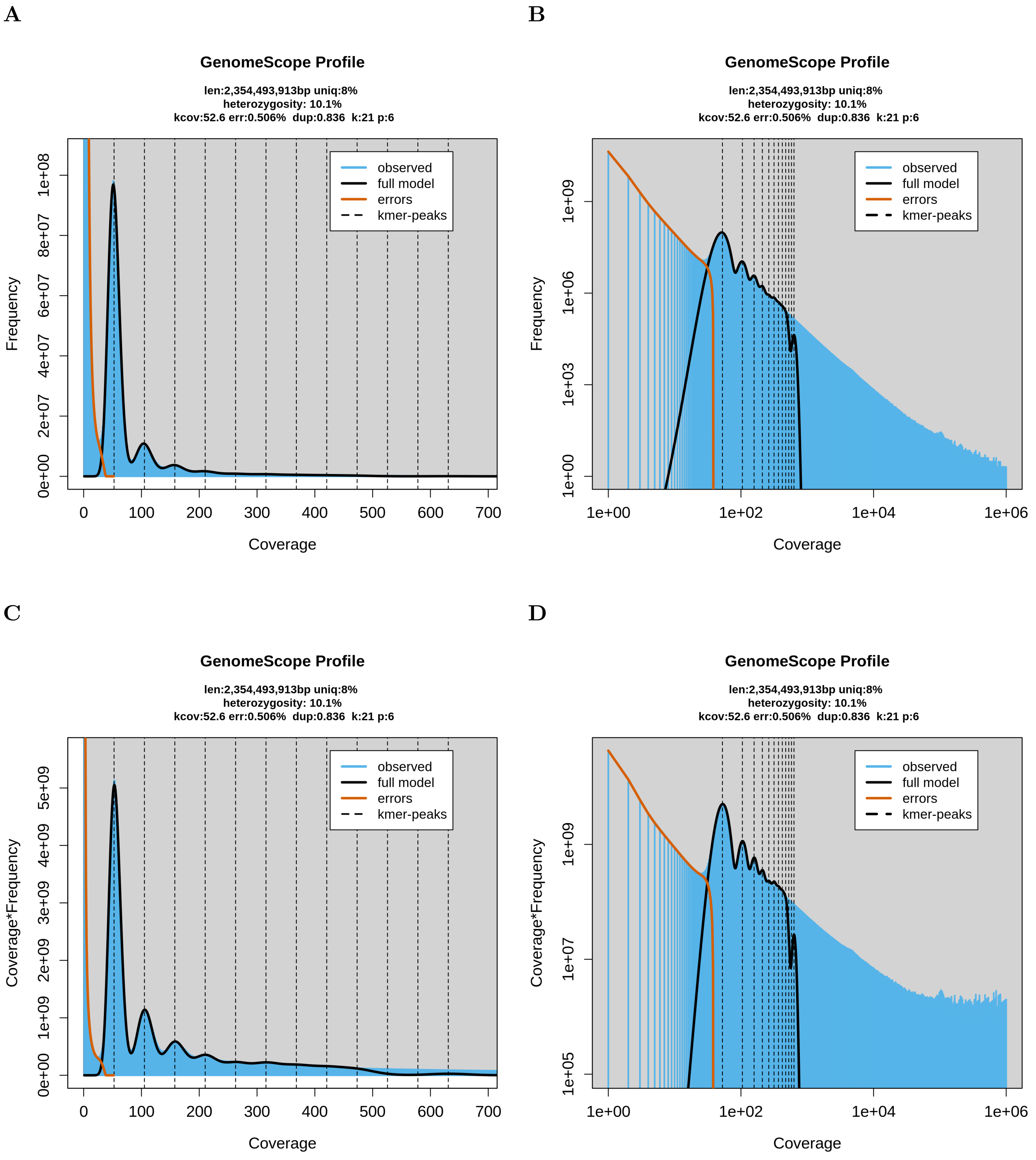

Supplementary Figure 21: GenomeScope wheat results (*Triticum aestivum*). Plots of the best fit model overlaying the k-mer spectrum for (A) untransformed linear, (B) untransformed log, (C) transformed linear, and (D) transformed log.

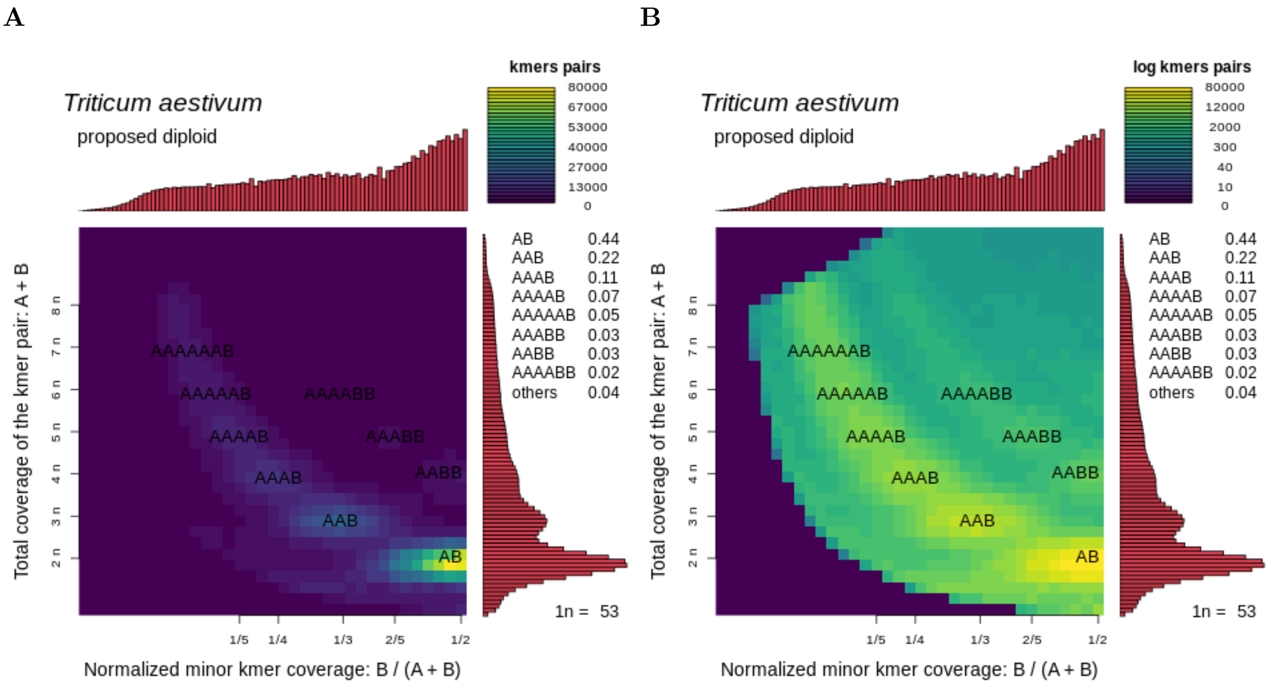

Supplementary Figure 22: Smudgeplot wheat results (*Triticum aestivum*). Smudgeplots are shown using either (A) a linear scale or (B) a log scale. The coloration indicates the approximate number of k-mer pairs per bin.

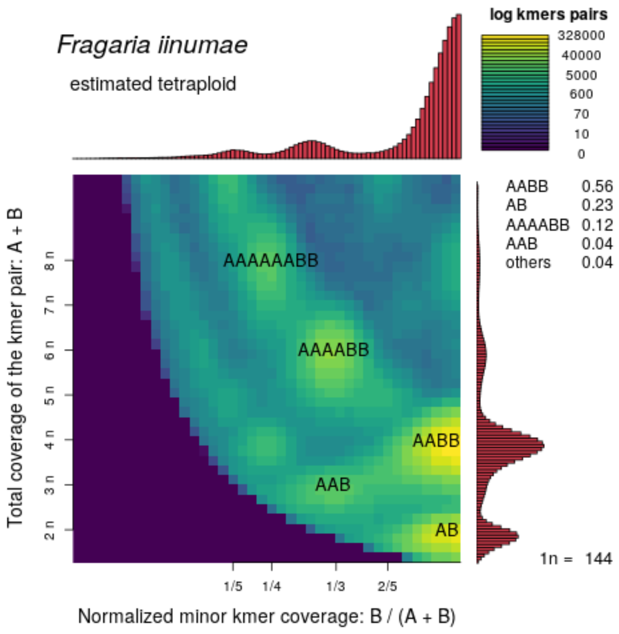

Supplementary Figure 23: Smudgeplot diploid strawberry results (*Fragaria iinumae*). Smudgeplot is shown using a log scale. The coloration indicates the approximate number of k-mer pairs per bin.

Supplementary Tables

Supplementary Table 1: Summary of polyploid genomes analyzed

Common Name	Species Name	SRA	Ploidy	Assembly Size
coastal redwood	*Sequoia sempervirens* (Save the Redwood Leagues 2019)	SRR9087413<br>SRR9087414<br>SRR9087417<br>SRR9087419<br>SRR9087420<br>SRR9087425<br>SRR9087426<br>SRR9087428<br>SRR9087450<br>SRR9087484<br>SRR9087485<br>SRR9087486<br>SRR9087487<br>SRR9087508<br>SRR9087509<br>SRR9087510<br>SRR9087511<br>SRR9087512<br>SRR9087516<br>SRR9087517<br>SRR9087528<br>SRR9087529<br>SRR9087530<br>SRR9087531<br>SRR9087532<br>SRR9087533<br>SRR9087534<br>SRR9087535<br>SRR9087536<br>SRR9087537	6	26.5 Gbp
cotton	*Gossypium barbadense* (Wang et al. 2019)	SRR1919013	4	2.267 Gbp
cotton	*Gossypium hirsutum* (Wang et al. 2019)	SRX4734214	4	2.347 Gbp
marbled crayfish	*Procambarus virginalis* (Gutekunst et al. 2018)	SRR5115143<br>SRR5115144<br>SRR5115145<br>SRR5115146<br>SRR5115147<br>SRR5115148	3	3.3 Gbp

root-knot nematode	*Meloidogyne arenaria* (Szitenberg et al. 2017)	SRR4242457 SRR4242468 SRR4242476 SRR4242477	4	163.7 Mbp
root-knot nematode	*Meloidogyne enterolobii* (Szitenberg et al. 2017)	SRR4242472 SRR4242473	3	162.4 Mbp
root-knot nematode	*Meloidogyne floridensis* (Szitenberg et al. 2017)	SRR4242474 SRR4242475	3	74.9 Mbp
root-knot nematode	*Meloidogyne incognita* (Szitenberg et al. 2017)	SRR4242460 SRR4242461	3	122.0 Mbp
root-knot nematode	*Meloidogyne javanica* (Szitenberg et al. 2017)	SRR4242458 SRR4242459	4	142.6 Mbp
potato	*Solanum tuberosum* (Hardigan et al. 2016)	SRR5349579	4	778.7 Mbp
wheat	*Triticum aestivum* (Zimin et al. 2017)	SRX2994097	6	15.34 Gbp

The assembly size refers to the size of the assembly presented in the corresponding cited work. The coastal redwood assembly size is reported at `https://nealelab.ucdavis.edu/redwood-genome-project-rgp/`.

Supplementary Table 2: Summary of estimated genome characteristics for polyploid species

**Species Name**	**Genome Size**	**Heterozygosity**	**Repetitiveness**
Sequoia sempervirens	27.0 Gbp	4.4%	53.5%
Gossypium barbadense	2.293 Gbp	11.6%	75.8%
Gossypium hirsutum	2.349 Gbp	11.8%	75.1%
Procambarus virginalis	9.5 Gbp	2.3%	81.1%
Meloidogyne arenaria	290.4 Mbp	8.0%	36.2%
Meloidogyne enterolobii	268.7 Mbp	6.1%	38.1%
Meloidogyne floridensis	201.7 Mbp	2.8%	24.4%
Meloidogyne incognita	207.4 Mbp	6.4%	29.2%
Meloidogyne javanica	280.2 Mbp	8.4%	35.1%
Solanum tuberosum	3.0 Gbp	6.9%	57.0%
Triticum aestivum	14.1 Gbp	10.1%	92.0%

GenomeScope 2.0 estimates for genome size, heterozygosity, and repetitiveness are shown for real sequencing data from 11 polyploid species. Genome size refers to the polyploid genome size. Heterozygosity refers to the nucleotide divergence. Repetitiveness refers to the percentage of the monoploid genome that consists of repetitive sequence.

Supplementary Table 3: Smudgeplot results on simulated polyploid data with heterozygosity sweep

Het.	Diploid	Triploid	Allotetraploid	Autotetraploid	Pentaploid	Hexaploid
0.5%	2	3	4	4	5	6
1.0%	2	3	4	4	5	6
1.5%	2	3	4	4	5	6
2.0%	2	3	4	4	5	6
2.5%	2	3	4	4	5	6
3.0%	2	3	4	4	5	6
3.5%	2	3	4	4	5	6
4.0%	2	3	4	4	5	6
4.5%	2	3	4	4	5	6
5.0%	2	3	4	4	5	6
5.5%	2	3	4	4	5	6
6.0%	2	3	4	4	5	6
6.5%	2	3	4	4	5	6
7.0%	2	3	4	4	5	6
7.5%	2	3	4	4	5	6
8.0%	2	3	4	4	5	6
8.5%	2	3	4	4	5	6
9.0%	2	3	4	4	5	6
9.5%	2	3	4	4	5	6
10.0%	4	3	4	4	5	6
10.5%	2	3	4	4	5	6
11.0%	2	3	4	4	5	6
11.5%	2	3	4	4	5	6
12.0%	2	3	4	4	5	6
12.5%	2	3	4	4	5	6
13.0%	2	3	4	4	5	6
13.5%	2	3	4	4	5	6
14.0%	2	3	4	4	5	6
14.5%	2	3	4	4	5	6
15.0%	2	3	4	4	5	6
15.5%	2	3	4	4	5	6
16.0%	2	3	4	4	5	6
16.5%	2	3	4	4	5	6
17.0%	2	3	4	4	5	6
17.5%	2	3	4	4	5	6
18.0%	2	3	4	4	5	6
18.5%	2	3	2	4	5	6
19.0%	2	3	2	4	5	6
19.5%	2	3	2	4	5	6
20.0%	2	3	2	4	5	6
20.5%	2	3	2	4	5	6
21.0%	2	3	2	4	5	6
21.5%	2	3	2	4	5	6
22.0%	2	3	2	4	5	6

22.5%	2	3	2	4	5	6
23.0%	2	3	2	4	5	6
23.5%	2	3	2	4	5	6
24.0%	2	3	2	3	5	6
24.5%	2	2	2	3	4	5
25.0%	2	2	2	3	4	5

Each column corresponds to the simulated ploidy, each row corresponds to the simulated heterozygosity, and each entry corresponds to the ploidy estimated by Smudgeplot. Smudgeplot is accurate over a wide range of heterozygosity values, only underestimating ploidy for extremely high heterozygosity values.

Supplementary Table 4: Smudgeplot results on simulated polyploid data with repetitiveness sweep

Rep.	Diploid	Triploid	Allotetraploid	Autotetraploid	Pentaploid	Hexaploid
1%	2	3	4	4	5	6
2%	2	3	4	4	5	6
3%	2	3	4	4	5	6
4%	2	3	4	4	5	6
5%	2	3	4	4	5	6
6%	2	3	4	4	5	6
7%	2	3	4	4	5	6
8%	2	3	4	4	5	6
9%	2	3	4	4	5	6
10%	2	3	4	4	5	6
11%	2	3	4	4	5	6
12%	2	3	4	4	5	6
13%	2	3	4	4	5	6
14%	2	3	4	4	5	6
15%	2	3	4	4	5	6
16%	2	3	4	4	5	6
17%	2	3	4	4	5	6
18%	2	3	4	4	5	6
19%	2	3	4	4	5	6
20%	2	3	4	4	5	6
21%	2	3	4	4	5	6
22%	2	3	4	4	5	6
23%	2	3	4	4	5	6
24%	2	3	4	4	5	6
25%	2	3	4	4	5	6
26%	2	3	4	4	5	6
27%	2	3	4	4	5	6
28%	2	3	4	4	5	6
29%	2	3	4	4	5	6
30%	2	3	4	4	5	6
31%	2	3	4	4	5	6
32%	2	3	4	4	5	6
33%	2	3	4	4	5	6
34%	2	3	4	4	5	6
35%	2	3	4	4	5	6
36%	2	3	4	4	5	6
37%	2	3	4	4	5	6
38%	2	3	4	4	5	6
39%	2	6	4	8	5	6
40%	4	6	4	8	5	6
41%	2	6	4	8	5	6
42%	4	6	4	8	5	6
43%	4	6	4	8	5	6
44%	4	6	8	8	5	6

45%	4	6	8	8	5	6
46%	4	6	8	8	5	6
47%	4	6	8	8	5	6
48%	4	6	8	8	5	6
49%	4	6	8	8	5	6
50%	4	6	8	4	5	6

Each column corresponds to the simulated ploidy, each row corresponds to the simulated repetitiveness, and each entry corresponds to the ploidy estimated by Smudgeplot. Smudgeplot is accurate over a wide range of repetitiveness values, only overestimating ploidy for extremely high repetitiveness values.

Supplementary Methods

Partially Ordered Sets

A partially ordered set, or *poset*, consists of a set X together with a binary relation $\leq$ satisfying reflexivity, anti-symmetry, and transitivity. Reflexivity states that for all $x \in X$, $x \leq x$. Anti-symmetry states that for all $x, y \in X$, $x \leq y$ and $y \leq x$ implies $x = y$. Transitivity states that for all $x, y, z \in X$, $x \leq y$ and $y \leq z$ implies $x \leq z$. A poset can be visualized by a directed acyclic graph in which the elements of the set are nodes in the graph and a directed edge exists from x to y if $x \leq y$. To simplify this graph, it is common practice to depict only the direct edges and to ignore edges that can be implied by the transitive property.

Common examples of a poset include the real numbers with the standard less-than-or-equal relation, the integers with the divisibility relation, and the powerset of a set with the inclusion relation. An example of a poset with the inclusion relation is shown in **Supplementary Figure 24**.

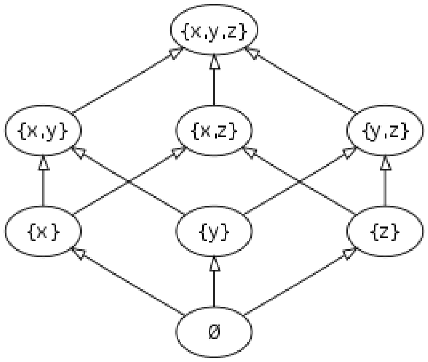

Supplementary Figure 24: Inclusion poset on the set $\{x, y, z\}$.

Integer Partitions

For GenomeScope 2.0, we use the poset on integer partitions with the refinement relation. An integer partition of a positive integer n is a unordered tuple of positive integers such that their sum equals n. For example, $(3, 1, 1, 1)$ is an integer partition of 6. We let $\Phi(n)$ denote the set of all integer partitions of n. We say that an integer partition φ is a refinement of the integer partition φ' if φ can be obtained by further partitioning elements of φ', and we denote this by $\varphi \leq \varphi'$. For example, $(1, 1, 1, 1, 1, 1) \leq (3, 1, 1, 1)$ because the element 3 can be partitioned into $(1, 1, 1)$. The poset of the integer partitions of 4 is shown in **Supplementary Figure 25**.

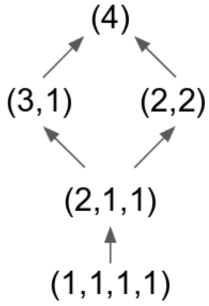

Supplementary Figure 25: Poset of the integer partitions of 4.

Möbius Inversion Formula on Integer Partitions

Let $s : \Phi(n) \to \mathbb{R}$ and $t : \Phi(n) \to \mathbb{R}$ be real-valued functions defined on the integer partitions of n, with the property that $t(\varphi) = \sum_{\varphi':\varphi\leq\varphi'} s(\varphi')$. Furthermore, assume that calculating $t(\varphi)$ is straightforward, but that we are actually interested in calculating $s(\varphi)$. The Möbius inversion formula allows us to invert the above equation to calculate $s(\varphi)$ in terms of $t(\varphi)$:

$$s(\varphi) = \sum_{\varphi':\varphi\leq\varphi'} \mu(\varphi,\varphi')t(\varphi') \tag{1}$$

where μ is the Möbius function. The Möbius function is defined as

$$\begin{aligned} \mu(\varphi,\varphi') &= 0 \text{ if } \varphi \nleq \varphi' \\ \mu(\varphi,\varphi) &= 1 \text{ for all } \varphi \in \Phi(n) \\ \mu(\varphi,\varphi') &= -\sum_{\varphi'':\varphi\leq\varphi''<\varphi'} \mu(\varphi,\varphi'') \text{ for } \varphi < \varphi' \end{aligned} \tag{2}$$

One useful property of Möbius functions is that they are defined based entirely on the poset structure, and are completely independent of the functions s and t.

Nucleotide Partitions

Recall the GenomeScope 2.0 polyploid model:

$$f(x) = G\sum_{i=1}^{2p} \alpha_i NB(x, i\lambda, \frac{i\lambda}{\rho}) \tag{3}$$

Now that we have introduced the necessary combinatorics theory, we more explicitly define the problem of determining α_i in terms of the ploidy, repetitiveness, heterozygosity, and k-mer length. Let the ploidy p be the number of sets of homologous chromosomes. We assume that for each of the chromosomes in a single complete set, all of the p corresponding homologues have exactly the same length.

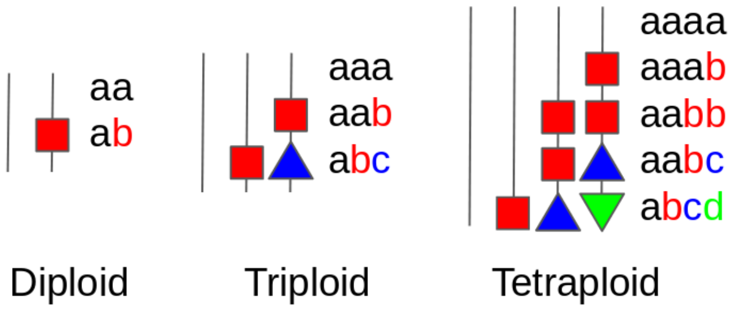

Supplementary Figure 26: Nucleotide heterozygosity forms for the diploid, triploid, and tetraploid cases. The black vertical lines refer to the homologous chromosomes. The colored shapes correspond to distinct mutations that have accumulated on the homologues.

For any given position along the genome, the p nucleotides at that position may be homozygous or heterozygous (see **Supplementary Figure 26**). In the diploid case, this corresponds to the

nucleotides being all the same, aa, or the nucleotides being all different, ab. These correspond to the integer partitions (2) and $(1,1)$ respectively. In the polyploid case, however, there are more complicated possibilities. For example, in the triploid case it is possible for two nucleotides to be the same and the third to be different, aab, corresponding to the integer partition $(2,1)$.

In general, the nucleotides may group according to any of the integer partitions of p. Furthermore, the order of a nucleotide partition doesn't matter, so aba and aab are equivalent. Indeed, this makes sense for our problem since the data in a k-mer spectrum are not homolog-specific and it is mathematically impossible to distinguish between equivalent nucleotide partitions.

Nucleotide Heterozygosity Rates

For our model we make the following assumptions: 1) each locus of the genome is independent of the other loci and 2) the nucleotide heterozygosity rates are constant over the entire genome. Unlike the infinite sites model, our model does not assume that every novel mutation must occur at a new site. With these assumptions we define nucleotide heterozygosity rates corresponding to the probabilities that the nucleotides across the p homologues at a given location of the genome partition according to a given integer partition. We define r_φ as the nucleotide heterozygosity rate corresponding to the nucleotide partition φ. For example, in the diploid case, the nucleotide heterozygosity rate, $r_{(1,1)}$, corresponds to the probability that the two nucleotides at a given position in the genome are distinct, i.e. that they partition according to ab. The nucleotide homozygosity rate, $r_{(2)}$, corresponds to the probability that the two nucleotides partition according to aa and is given by $r_{(2)} = 1 - r_{(1,1)}$.

Similarly, in the polyploid case, the nucleotide heterozygosity rates are defined according to the nucleotide partitions. For example, in the hexaploid case, $r_{(3,2,1)}$ corresponds to the probability that the nucleotides partition according to $aaabbc$. The nucleotide homozygosity rate, $r_{(6)}$, corresponds to the probability that the nucleotides partition according to $aaaaaa$, and is given by $1 - \sum_{\varphi' < (6)} r_{\varphi'}$. These nucleotide homozygosity rates are the parameters that are estimated by GenomeScope 2.0 through the non-linear optimization algorithm.

K-mer Partitions

As the k-mer spectrum deals with k-mers and not with individual nucleotides, it is necessary to relate nucleotide heterozygosity rates with k-mer partition rates. Let k correspond to the k-mer length. Note that for any position along the genome (except for the final $k-1$ positions on each chromosome), the p k-mers beginning at this position may group according to any of the integer partitions of p. Similar to nucleotide partitions, the order of k-mer partitions doesn't matter, so ABA is equivalent to AAB. Furthermore, as with nucleotide partitions, it is mathematically impossible to distinguish between equivalent k-mer partitions in the k-mer spectrum.

K-mer Heterozygosity Rates

We define k-mer heterozygosity rates corresponding to the probabilities that the k-mers across the p homologues at a given location of the genome partition according to a given integer partition. We define s_φ as the k-mer heterozygosity rate corresponding the the k-mer partition φ. In the diploid case, the k-mer partition rates $s_{(2)}$ and $s_{(1,1)}$ correspond to the probabilities that the two k-mers at a given position (in a non-repetitive region of the genome) partition according to AA and AB respectively. Note that the only way for the k-mers to partition according to AA is if, for each of the k positions along the k-mer, the nucleotides partition according to aa (see **Supplementary**

Figure 27). Thus, with our model assumptions, $s_{(2)} = (r_{(2)})^k$, which is equivalent to the more general form:

$$\sum_{\varphi':(2)\leq\varphi'} s_{\varphi'} = \left(\sum_{\varphi':(2)\leq\varphi'} r_{\varphi'} \right)^k \tag{4}$$

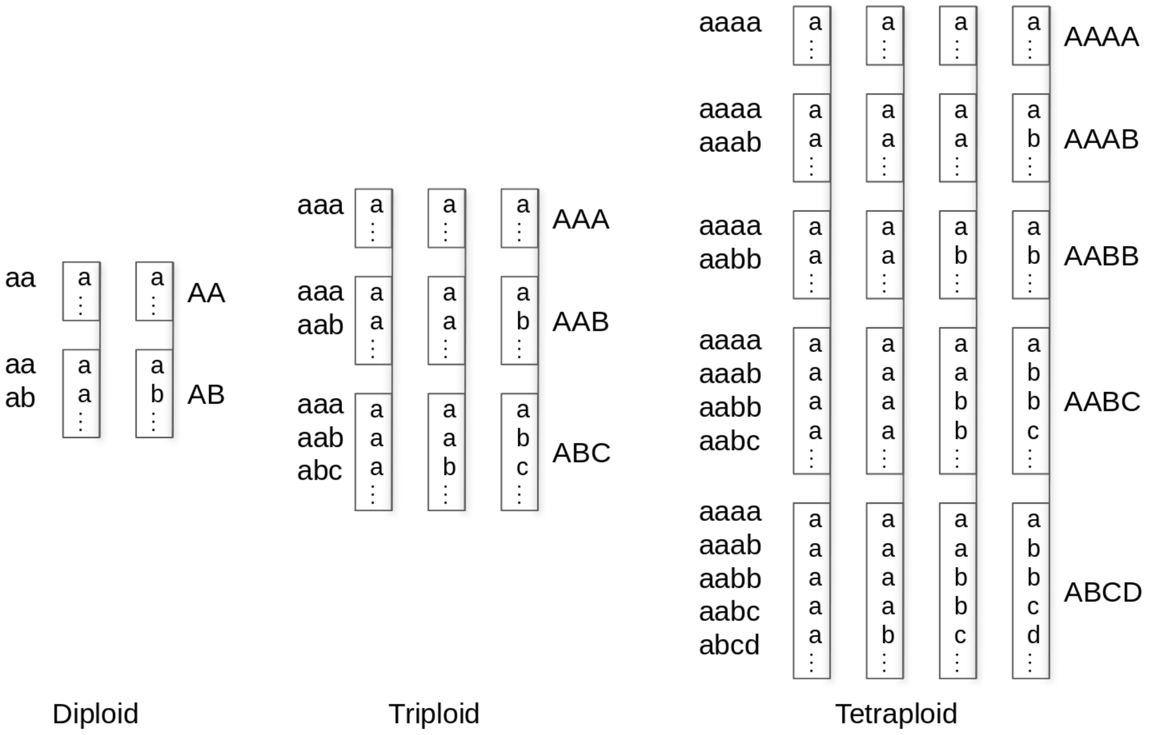

Supplementary Figure 27: K-mer heterozygosity forms and their corresponding nucleotide heterozygosity forms in the diploid, triploid, and tetraploid cases. The black vertical lines refer to the homologous chromosomes. The black boxes refer to the k-mers on the homologues. The nucleotide heterozygosity forms on the left are compatible with the k-mer heterozygosity form on the right. Specifically, the k-mers will partition according to the k-mer partition on the right, as long as they are made up of any combination of nucleotides partitioned according to the nucleotide heterozygosity forms on the left.

To determine $s_{(1,1)}$, one must consider which nucleotide partitions are compatible with the k-mer partition AB. In fact, both ab and aa are compatible. For example, consider the k-mers *gattaca* and *cattaca*. These k-mers are distinct and thus partition according to AB. However, while the nucleotides at the first position partition according to ab, the nucleotides at positions two through seven partition according to aa. Thus, $(r_{(1,1)} + r_{(2)})^k$, which represents the probability that the nucleotides at every position along the k-mer partition according to ab or aa, is equivalent to the probability that the k-mers partition according to AB or AA. This yields

$$s_{(1,1)} + s_{(2)} = (r_{(1,1)} + r_{(2)})^k \tag{5}$$

which is equivalent to the more general form

$$\sum_{\varphi':(1,1)\le\varphi'} s_{\varphi'} = \left(\sum_{\varphi':(1,1)\le\varphi'} r_{\varphi'}\right)^k \tag{6}$$

This further implies

$$s_{(1,1)} = (r_{(1,1)} + r_{(2)})^k - s_{(2)} = (r_{(1,1)} + r_{(2)})^k - (r_{(2)})^k = 1 - (r_{(2)})^k \tag{7}$$

In the general polyploid case, it is possible to determine which nucleotide partitions are compatible with a given k-mer partition by using the integer partition poset. Specifically, any nucleotide partition φ in the poset is compatible with any k-mer partition φ' in the poset if and only if $\varphi \ge \varphi'$. For example, returning to *gattaca* and *cattaca*, we have that *aa* is compatible with AB since $(2) \ge (1,1)$.

Let $t_\varphi = \sum_{\varphi':\varphi\le\varphi'} s_{\varphi'}$ represent the probability that the k-mers partition according to φ or any other partition φ' with $\varphi < \varphi'$. This is straightforward to calculate in terms of nucleotide partition rates as $t_\varphi = \left(\sum_{\varphi':\varphi\le\varphi'} r_{\varphi'}\right)^k$.

Applying the Möbius Inversion Formula

Using the Möbius inversion formula, we can calculate s_φ in terms of t_φ. Specifically, we have

$$s_\varphi = \sum_{\varphi':\varphi\le\varphi'} \mu(\varphi,\varphi') t_{\varphi'} = \sum_{\varphi':\varphi\le\varphi'} \mu(\varphi,\varphi') \left(\sum_{\varphi'':\varphi'\le\varphi''} r_{\varphi''}\right)^k \tag{8}$$

which gives us the k-mer heterozygosity rates in terms of the nucleotide heterozygosity rates.

K-mer Frequency Contributions in Non-Repetitive Regions

With these equations derived for the k-mer partition rates, it is necessary to determine how the p k-mers of each of the possible k-mer partitions contribute to the $2p$ peaks of the k-mer spectrum. Let $M_i(\varphi)$ denote the frequency contribution to peak i by the p k-mers (in a non-repetitive region) partitioned according to φ. For example, if $\varphi = AAABBCCD$, then $M_1(\varphi) = 1$ because the D k-mer contributes to the first peak, $M_2(\varphi) = 2$ since the B and C k-mers contribute to the second peak, and $M_3(\varphi) = 1$ since the A k-mer contributes to the third peak.

K-mer Frequency Contributions in Repetitive Regions

For k-mers that are a two-copy repeat, there are two locations of the genome where they occur. Let φ_1 be the k-mer partition of the p k-mers at the first location, and φ_2 be the k-mer partition of the p k-mers at the second location. We make the simplifying assumption that the repetitive k-mer (i.e. the k-mer that is equivalent between the two k-mer partitions) is the most prevalent k-mer in each of the two k-mer partitions. For example, if $\varphi_1 = AAABBC$ and $\varphi_2 = AABBCC$, then the overall k-mer partition of the $2p$ k-mers is $AAAAABBCCDDE$. Specifically, we consider the A k-mers between partitions to be equivalent, but not the B and C k-mers. Then, we may let $M_i(\varphi_1, \varphi_2)$ denote the frequency contribution to peak i by the $2p$ k-mers (in a two-copy repeat) partitioned according to φ_1 and φ_2.

Polyploid Alpha Coefficients

Finally, we have:

$$\alpha_i = (1-d) \sum_{\varphi \in \Phi(p)} M_i(\varphi) s_\varphi + d \sum_{\varphi_1 \in \Phi(p)} \sum_{\varphi_2 \in \Phi(p)} M_i(\varphi_1, \varphi_2) s_{\varphi_1} s_{\varphi_2} \tag{9}$$

where d is the proportion of distinct k-mers of the monoploid genome that occur twice, p is the ploidy, $\Phi(p)$ is the set of integer partitions of p, $M_i(\varphi)$ and $M_i(\varphi_1, \varphi_2)$ are the frequency contributions to peak i of the k-mers partitioned according to φ or (φ_1, φ_2) respectively, and s_φ is the k-mer heterozygosity rate of the k-mer partition φ. For each ploidy up to $p = 6$, we have explicitly written in the code the many terms for the equations for α_i and s_φ. Then, non-linear optimization is used to determine the parameters that minimize the residual sum of squares between the model and the real data. GenomeScope 2.0 currently only supports analyzing organisms with ploidy up to 6, due to the combinatorial number of terms in these equations.

Smudge Annotation

The annotation of smudges (pseudocode below) consist of three steps: 1) identification of smudge boundaries, 2) smudge filtering and 3) estimation of monoploid coverage. First, the 2D space is divided into bins and the number of k-mer pairs in each bin is calculated. Then, the centers of each smudge are chosen to be the bins corresponding to local maxima (in terms of the number of k-mer pairs). The k-mer pairs in all the other bins are aggregated to the nearest neighbouring bin that is designated as a smudge center. Once the boundaries of individual smudges are estimated, we filter smudges that represent less than 0.5% of the dataset (i.e. they contain less than 0.5% of the k-mer pairs), as these usually represent repetitive structures of the genome and are frequently misplaced due to too few k-mers representing them.

For the first estimation of the monoploid coverage, we calculate an estimate for each of the identified smudges, and then calculate an overall estimate as the weighted mean of these estimates where the weights are the number of k-mer pairs within each smudge. To calculate the estimate for an individual smudge, we first label the smudge according to its putative structure. For example, of all the smudges with a relative minor coverage near 0.5, the one with the lowest sum of coverages is assumed to be AB and others are labeled using the AB smudge as a reference. This process is continued for all relative minor coverages of the identified smudges until all smudges are labeled. Finally, the estimate of monoploid coverage for an individual smudge is given by its sum of coverages divided by the number of k-mers that make up its labeled structure. For example, the estimate for an AAB smudge would be $\frac{CovA+CovB}{3}$ since three k-mers make up the AAB structure.

Next, this first estimate of monoploid coverage is used to re-annotate smudges and subsequently to estimate the ploidy. If multiple smudges get annotated with the same genome structure, the whole process is repeated with lowered resolution (i.e. the number of bins in the 2D plot is decreased). This estimate of monoploid coverage assumes that we correctly labeled each smudge with its putative structure, which may not be the case if we didn't correctly find the smudge with lowest sum of coverages for a given relative minor coverage. Therefore, the final estimate of monoploid coverage is refined by using kernel smoothing applied on the subset of k-mer pairs within the brightest smudge in the Smudgeplot.

Pseudocode for λ Estimation

Algorithm 1 Calculate λ

for all $expected\_minor\_coverage \in (\frac{1}{2}, \frac{1}{3}, \frac{1}{4}, \frac{1}{5}, \frac{1}{6})$ **do**
  kmer_pairs_subset = all kmer pairs with $|minor\_coverage - expected\_minor\_coverage| \leq 0.01$
  peaks = find_local_maxima_using_kernel_smoothing(coverage_sums_of_kmer_pairs_subset)
  for all peak in peaks **do**
    λ_peak_est = $exp\_min\_cov * \frac{peak\_cov}{round(\frac{peak\_cov}{min(peak\_covs)})}$
  end for
end for
λ = weighted_mean(λ_peak_ests, peak_weights)

Topologies

In the field of phylogenetics, the evolutionary relationships between species are often depicted in a branching diagram known as a phylogenetic tree. In this setting, the topology of the tree refers to the branching structure of the tree. We may also depict the similarities between homologous chromosomes in a branching diagram. In this case, a topology refers to the similarities between distinct homologues.

For ploidies of 4 and greater, there are multiple possible topologies (see **Supplementary Figure 28**). For example, the two tetraploid topologies as notated in Newick notation are $(4,(3,(2,1)))$; and $((4,3),(2,1))$;.

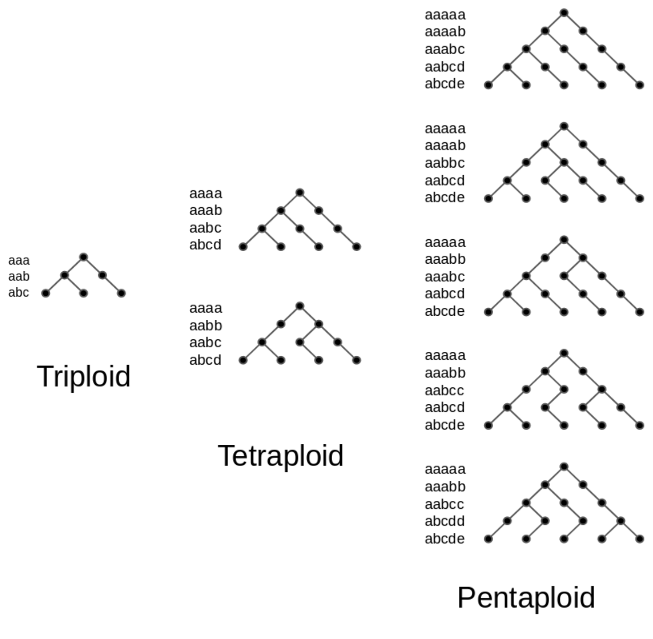

Supplementary Figure 28: Topologies in the triploid, tetraploid, and pentaploid cases. To the left of each tree are the nucleotide heterozygosity forms that are compatible with that tree.

For an autotetraploid organism, a whole genome duplication event has occurred sometime in its evolutionary history. Thus, for a given locus, the two k-mers at this locus of the ancestral genome were either heterozygous or homozygous (for an ancestral mutation) at the time of duplication. If

the ancestral k-mers were homozygous at this locus, then the four k-mers of the polyploid organism immediately after the duplication were of the form AAAA.

Now we must consider the possibility that a more recent mutation that overlaps the k-mers at this locus has accumulated in the population. In this case, after recombination a sequenced individual may have this new mutation in zero, one, two, three, or four homologues. If this new mutation occurs in one or three homologues, then the k-mers are of the form AAAB. If this new mutation occurs in two homologues, then the k-mers are of the form AABB. Notably, AAAB is more prevalent than AABB because it is more likely that a mutation will be on any one homologue or any three homologues $(4p(1-p)^3 + 4p^3(1-p))$ versus any two homologues $(6p^2(1-p)^2)$, where p is the allele frequency of the mutation in the population.

If instead the ancestral k-mers were heterozygous at this locus (which is rarer than the k-mers being homozygous at this locus), then the four k-mers of an ancient polyploid organism immediately after duplication were of the form AABB. For a modern organism which has undergone recombination, this ancestral mutation may be present in any number of the four homologues.

If the ancestral mutation is present in zero or all four homologues, then the k-mers (disregarding modern mutations) are of the form AAAA. Again, we must then consider that a more recent mutation may be present in any number of homologues of a sequenced individual. If the recent mutation is present in one or three homologues, then the k-mers are of the form AAAB, while if it is present in two homologoues, then the k-mers are of the form AABB. Again, AAAB would be more prevalent than AABB due to the same reasoning as above.

Finally, if the ancestral mutation were present in one or three homologues, then the k-mers were of the form AAAB, while if it were present in two homologues, then the k-mers were of the form AABB. Again, AAAB would be more prevalent than AABB. In summary, we would expect that the prevalence of AAAB would be much greater than the prevalence of AABB in autotetraploid species.

Intuitively, the only ways for the k-mers to partition according to AABB in an autotetraploid species are 1) the k-mers were homozygous before the duplication event and any modern mutations have accumulated on exactly two homologues after recombination or 2) the k-mers were heterozygous before the duplication event and the the ancient mutation has accumulated on exactly two homologues after recombination and any modern mutation has accumulated on the same two homologues or on the opposite two homologues. For this reason, we would expect that the k-mer heterozygosity rate of AABB in autotetraploid species lower than that of AAAB, and define the "autotetraploid topology" as $(4,(3,(2,1)))$; which corresponds to the heterozygosity forms $AAAA$, $AAAB$, $AABC$, and $ABCD$.

For an allotetraploid organism, two similar but distinct ancestral species have undergone a hybridization event sometime in its evolutionary history. Thus, for a given locus, the two k-mers of the first ancestral genome may either be heterozygous or homozygous (for an ancestral mutation) and the two k-mers of the second ancestral genome may either be heterozygous or homozygous (for another ancestral mutation). If the k-mers at this locus in both ancestral genomes were homozygous, which is quite likely, then we would expect the k-mers to be of the form AABB. Furthermore, due to the preferential chromosomal pairing of A with A and B with B that is often the case during meiosis with allotetraploid species, we would still expect a high prevalence of AABB after recombination.

Thus in the allotetraploid case, AABB is more prevalent because it is much more likely that the k-mers at a particular locus in the ancestral genomes were homozygous rather than heterozygous and because it is much more likely that homologous chromosomes from the same ancestral species pair together during meiosis. Intuitively, the reason why AABB is more prevalent for allotetraploid species than for autotetraploid species is because for allotetraploid species there are two distinct genomes. Thus, homozygous locations of the genome can result in AABB, whereas

for autotetraploid species there is only a single duplicate genome so homozygous locations necessarily result in AAAA. In this case AABB is then only possible for an autotetraploid species if a more recent mutation occurs in exactly two homologues. In summary, we would expect that the prevalence of AABB would be much greater than the prevalence of AAAB in allotetraploid species. For this reason, we define the "allotetraploid topology" as $((4,3),(2,1))$; which corresponds to the heterozygosity forms $AAAA$, $AABB$, $AABC$, and $ABCD$.
