# Supplementary material for: GenomeScope 2.0 and Smudgeplot for reference-free profiling of polyploid genomes
Source: Nat Commun. 2020 Mar 18;11:1432. doi: 10.1038/s41467-020-14998-3 (PMC7080791; doi:10.1038/s41467-020-14998-3)
Supplement: Supplementary file 4 — Description of Additional Supplementary Files [file 41467_2020_14998_MOESM4_ESM.pdf]

Title: Supplementary Data 1:
Description: Full GenomeScope results on simulated data. Results for triploid, tetraploid, pentaploid, and hexaploid simulated datasets are in separate sheets. Mean absolute errors are located at the bottom of each sheet.
